# Spatiotemporally Ultrasound‐Controlled Nanoparticles Reprogramming Immunostimulatory Antigen‐Presenting Cancer‐Associated Fibroblasts to Enhance Cancer Immunotherapy

**DOI:** 10.1002/advs.76673

**Published:** 2026-07-27

**Authors:** Chen Ai, Weikai Sun, Yuxuan Zhao, Daqian Sun, Ting Meng, Yafei Qi, Fengyang Jiang, Jintang Sun, Zhiliang Gao, Dexin Yu

**Affiliations:** ^1^ Department of Radiology Qilu Hospital of Shandong University Jinan Shandong People's Republic of China; ^2^ Translational Medicine Research Center in Nano Molecular and Functional Imaging of Shandong University Jinan People's Republic of China; ^3^ Research Center for Basic Medical Sciences Qilu Hospital of Shandong University Jinan People's Republic of China; ^4^ Shandong Key Laboratory of Magnetic Field‐Free Medicine & Functional Imaging Shandong University Jinan Shandong People's Republic of China; ^5^ Research Institute of Magnetic Field‐Free Medicine & Functional Imaging Shandong University Jinan Shandong People's Republic of China; ^6^ National Medicine‐Engineering Interdisciplinary Industry‐Education Integration Innovation Platform Shandong University Jinan Shandong People's Republic of China

**Keywords:** antigen‐presenting CAFs, cancer‐associated fibroblasts, immunostimulatory CAFs, nanoparticles, tumor immune microenvironment

## Abstract

Cancer‐associated fibroblasts (CAFs) are a major component of the tumor microenvironment and represent a potential therapeutic target for fibrotic breast cancer. Myofibroblastic CAFs (myCAFs), the major subtype, form a tumor barrier by generating a dense extracellular matrix (ECM) that greatly limits the penetration of immune cells and promotes an immunosuppressive microenvironment. Reprogramming the immunosuppressed myCAFs into the immune activated antigen presenting CAF (apCAFs), rather than directly eliminating CAFs, is a better treatment modality. We developed a spatiotemporally ultrasound‐controlled nanoparticle (mRNA/V@M NPs) based on a lipid‐polymer hybrid drug delivery platform to co‐encapsulate CD74 mRNA and V‐9302. Interestingly, this study found that successfully reprogrammed myCAFs could activate CD4^+^T cells, reduce the ECM, and promote immune cell infiltration through the CD74‐MHC II pathway. To overcome the limitation of MHC I immunodeficiency, V‐9302 was used to induce immunogenic cell death (ICD) in tumor cells and activate dendritic cells (DCs) to compensate for MHC I‐mediated immunodeficiency. In conclusion, in a fibrotic triple‐negative breast cancer mouse model, mRNA/V@M NPs combined with ultrasound treatment significantly enhanced MHC I and MHC II activation and increased CD8^+^ T cell infiltration and suppressed immunosuppressive TME, thereby triggering robust systemic antitumor immunity.

## Graphical abstract

We developed an ultrasound‐controlled biomimetic nanoplatform, mRNA/V@M NPs, to co‐deliver CD74 mRNA and V‐9302 for fibrotic TNBC therapy. CD74 mRNA reprograms myCAFs into antigen‐presenting apCAF cells through the CD74‐MHC II pathway, while V‐9302 induces ICD and activates MHC I‐CD8^+^ T cell immunity. This dual MHC II/MHC I immune activation remodels the fibrotic immunosuppressive TME and enhances antitumor efficacy.

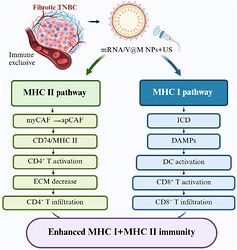

## 1 Introduction

The tumor microenvironment (TME) consists of diverse cellular and non‐cellular components that collectively drive tumor growth, invasion, metastasis, and immune evasion [1, 2, 3]. As one of the most abundant stromal cell populations within the TME, cancer‐associated fibroblasts (CAFs) play central roles in tumor progression through extracellular matrix (ECM) remodeling, paracrine signaling, and immune modulation [4, 5]. However, therapeutic strategies based on broad CAF depletion may disrupt stromal integrity and ECM architecture, thereby paradoxically accelerating tumor cell dissemination [6, 7]. This risk is closely associated with the pronounced heterogeneity and spatial organization of CAF subsets. Cancer cell‐adjacent myofibroblastic CAFs (myCAFs) produce collagen‐rich ECM and matrix remodeling programs that contribute to fibrosis and immune exclusion; although partial suppression of myCAF associated fibrosis may improve therapeutic penetration, complete myCAF elimination may collapse stromal barriers that physically restrain tumor cell invasion and metastatic escape [8, 9, 10, 11, 12]. Inflammatory CAFs (iCAFs), often enriched at the tumor periphery or in perivascular regions, regulate cytokine chemokine signaling, angiogenesis, and myeloid cell recruitment; thus, selective inhibition of tumor‐promoting inflammatory programs may relieve immunosuppression and therapeutic resistance [13]. In contrast, depletion of immune‐supportive MHC II^+^/CD74^+^ antigen presenting CAF subsets may disrupt tertiary lymphoid structures (TLS) associated and T cell enriched stromal niches, thereby compromising antitumor immunity [14]. These subtype and niche dependent consequences underscore the need to move beyond indiscriminate CAF ablation toward subtype‐specific modulation or functional reprogramming of CAFs into antitumor fibroblast states.

CAFs are highly heterogeneous and phenotypically plastic, encompassing both tumor promoting and tumor restraining fibroblast states [15, 16, 17, 18]. Among these subsets, apCAFs represent an immunologically active CAF state characterized by MHC class II associated antigen presenting features, including expression of the invariant chain CD74 [19, 20, 21, 22]. CD74 is required for MHC class II assembly and intracellular trafficking from the rough endoplasmic reticulum to late endosomal compartments, thereby supporting antigen presentation and CD4^+^ T cell immune activation [23]. However, CAF states are dynamically regulated during tumor progression. ApCAF cells may lose MHC II associated features and shift toward α‐SMA^+^ myCAF phenotypes, contributing to stromal fibrosis, immune exclusion, and impaired therapeutic delivery [20, 21, 24, 25, 26, 27, 28]. Conversely, restoring CD74 expression may reinforce MHC II mediated antigen presenting functions and promote CD4^+^ T cell dependent antitumor immunity [20, 29]. In this context, mRNA‐based gene delivery provides a transient and programmable approach to regulate CAF function without permanent genetic alteration [30, 31, 32]. Here, we propose a CD74 mRNA‐based strategy to reprogram tumor‐promoting myCAFs into immunologically active apCAF cells, aiming to convert CAFs from a physical and immunological barrier into an immune‐activating stromal platform while avoiding stromal ablation.

The absence of antigen and major histocompatibility complex class I (MHC‐I) expression on tumor cells undermines the efficacy of current therapeutic approaches. Consequently, there is a critical requirement for strategies that concurrently boost MHC‐I levels to flag tumor cells and reinstate T cell‐mediated recognition [33, 34]. Immunogenic cell death (ICD) has shown great potential for anti‐tumor immunity by triggering the MHC‐I pathway [35, 36]. V‐9302 (a glutamine transporter inhibitor)has been reported to highly activate the ICD effect [37, 38, 39, 40, 41], which causes tumor cells to release damage‐associated molecular patterns (DAMPs) such as calreticulin (CRT), high mobility group box 1 (HMGB1), and adenosine triphosphate (ATP) to activate dendritic cells (DCs) [42]. Thus, the MHC I signaling pathway promotes antigen presentation and activates CD8^+^ T cells. To translate CAF reprogramming and ICD induction into effective therapy, efficient and spatially controllable delivery of CD74 mRNA and V‐9302 into stromal and tumor cells is essential. However, the pre‐existing dense ECM barrier and insufficient tumor perfusion in fibrotic tumors severely restrict nanomedicine penetration and intracellular cargo delivery [41]. Ultrasound targeted microbubble destruction (UTMD) has emerged as a promising strategy for local drug and gene delivery, as acoustic cavitation and sonoporation can transiently increase vascular and membrane permeability, promote nanoparticle penetration, and enhance intracellular cargo release [43]. Therefore, coupling UTMD with a CD74 mRNA/V‐9302 co‐delivery nanoplatform may enable spatially controllable CAF reprogramming and ICD induction, thereby amplifying antitumor immune activation.

**SCHEME 1:**
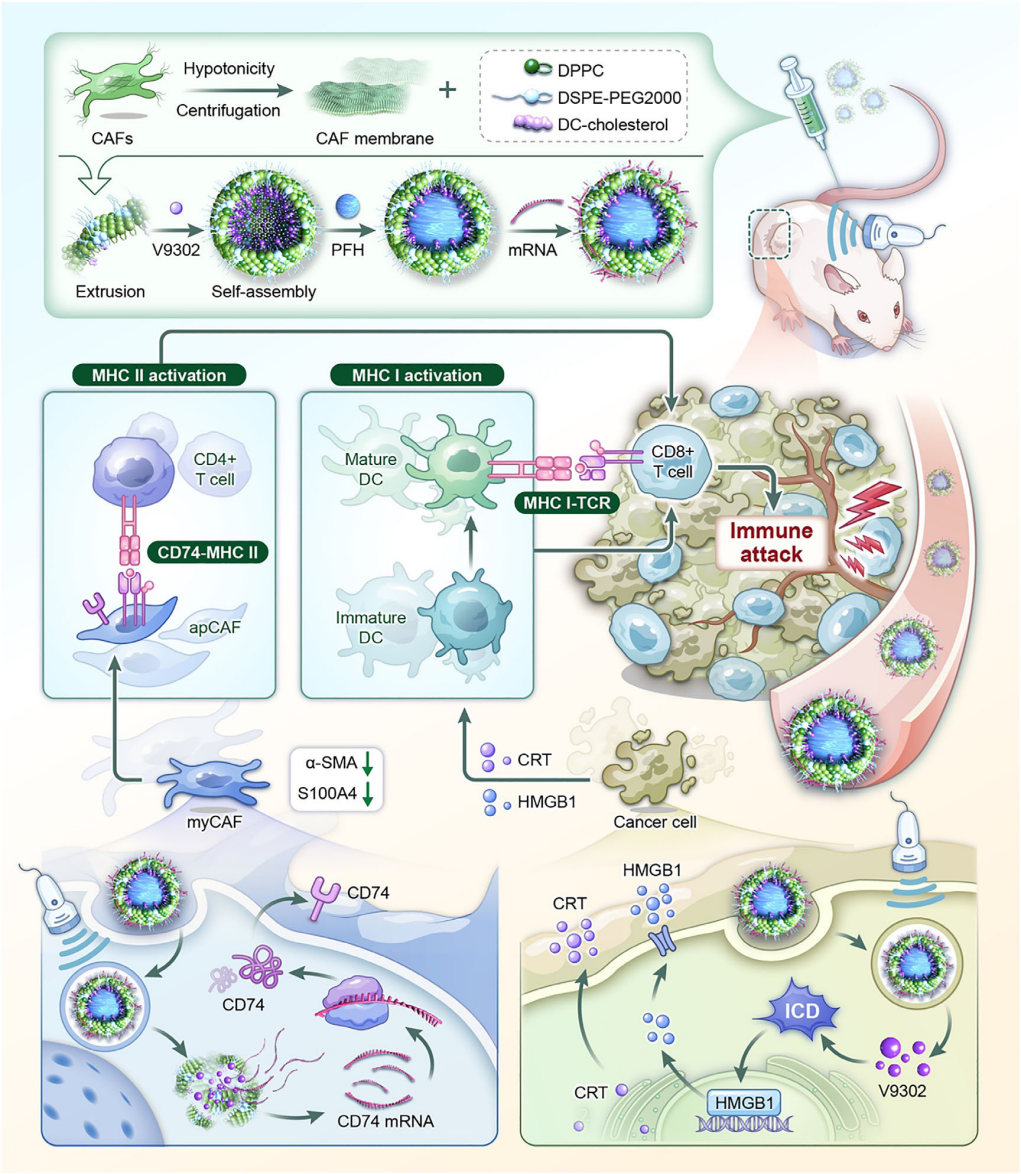
Schematic illustration of tumor ECM‐targeted of mRNA/V@M NPs combined with US to modulate the immune microenvironment for effective immunotherapy against fibrotic TNBC.

Based on the above considerations, we innovatively developed spatiotemporally ultrasound‐controlled nanoparticles (mRNA/V@M NPs) to reprogram the cancer‐promoting myCAF phenotype to the immune‐activated apCAF phenotype through the CD74‐MHC II pathway. Under the effect of UTMD, ultrasound‐mediated cavitation and sonoporation promoted the accumulation of CD74 mRNA and V‐9302 in tumor tissues, and the gene transfection efficiency was significantly improved. V‐9302 efficiently and effectively activated ICD effects through MHC I signaling. Interestingly, we further observed that the CD74‐MHC II pathway can facilitate the entry of CD8^+^ cells into breast cancer cells by disrupting the expression of myCAFs, a barrier on the tumor surface, which is currently the focus of research. Hence, the combination of MHC I and MHC II signaling pathways led to a significant increase in CD8^+^ cell infiltration, suppressing the immunosuppressive TME, conferring potential antitumor properties on triple‐negative breast cancer (TNBC) from immunologically “cold” to “hot” tumors. The composite membrane formed by the fusion of the CAFs membrane and lipid can realize the TME targeting of nanoparticles, and its bionic characteristics can achieve precise targeting and ensure the effective delivery of nanoparticles to the tumor site. In summary, we explored the feasibility of reprogramming fibroblast subtypes for cancer therapy, as well as synergistic MHC I and MHC II signaling pathways, an innovative strategy that improves immunity and exerts significant anticancer effects (Scheme 1).

## 2 Results and Discussion

### 2.1 Preparation and Characterization of mRNA/V@M

**FIGURE 1:**
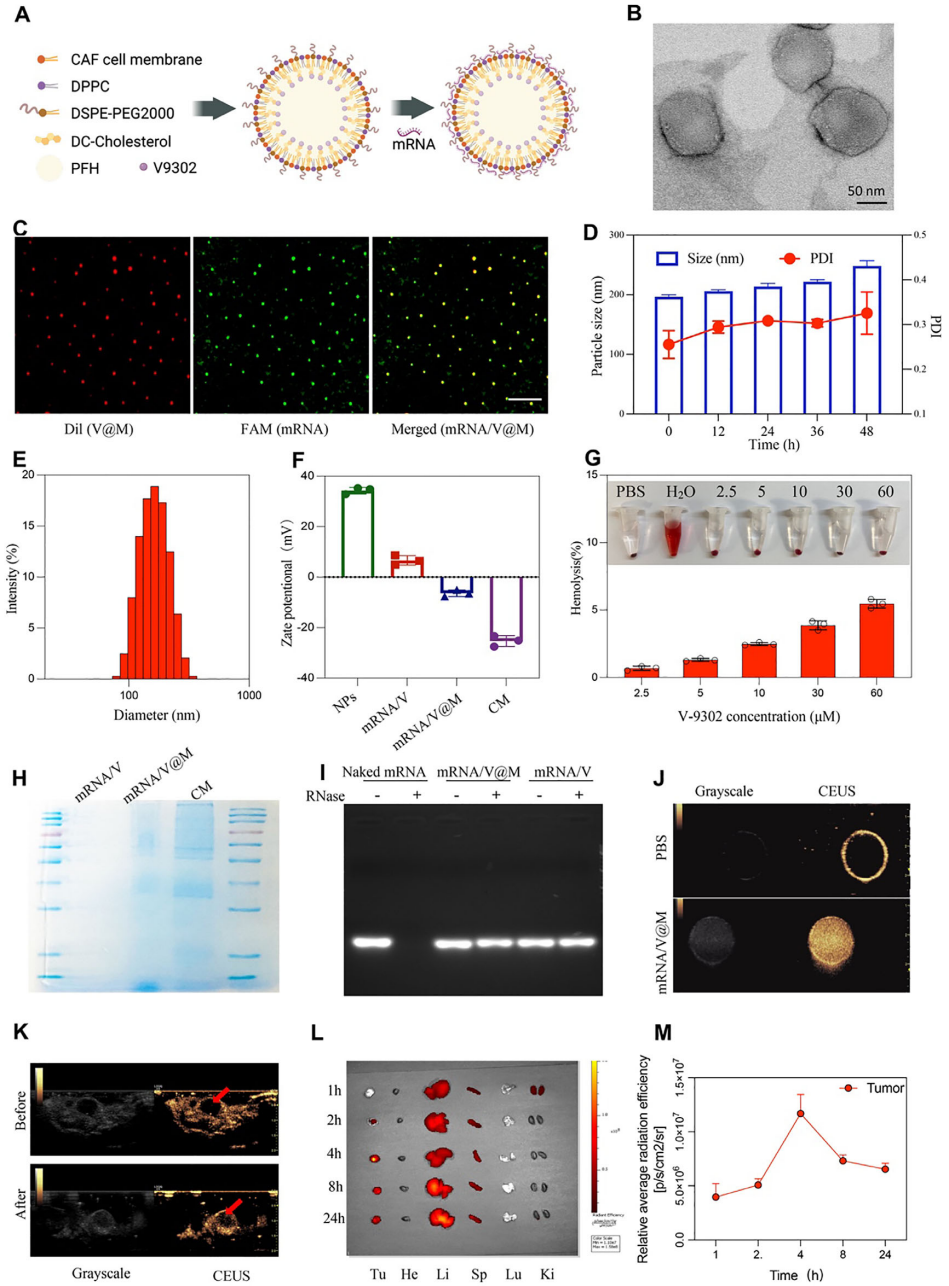
Preparation and characterization of mRNA/V@M nanoparticles. (A) The synthesis of mRNA/V@M (the schematic diagram was created by Biorender). (B) TEM image of mRNA/V@M. Scale bars, 50 nm. (C) CLSM images of V@M adsorbed FAM mRNA. Scale bars, 10 µm. (D) The size and PDI of mRNA/V@M were monitored by dynamic light scattering (DLS) for 48 h. n = 3. (E) Size distribution of mRNA/V@M. (F) Zeta potential of Blank NPs, mRNA/V, mRNA/V@M, and CM. n = 3. (G) Hemolytic rate comparison between different concentrations of V‐9302. (H) The presence of cell membrane coatings on mRNA/V@M complexes was assessed via gel electrophoresis. The gel configuration included: Lane 1:naked nanoparticles, Lane 2:cell membrane cloaked nanoparticles, and Lane 3 ‐ pure cell membrane preparation. (I) Stability of mRNA/V and mRNA/V@M incubated with (+) or without (−) RNase. (J) US imaging of grayscale and CEUS in vitro. (K) US imaging of grayscale and CEUS in vivo. The red arrow marks the position of the tumor. (L, M) Fluorescence imaging and quantitative evaluation of isolated tumors and major organs after tail vein injection of DiR‐labeled mRNA/V@M. Tu, He, Li, Sp, Lu, and Ki represent tumor, heart, liver, spleen, lung, and kidney, respectively. n = 3.

We prepared a lipid polymer hybrid drug delivery system (mRNA/V@M) with CAFs targeting properties (Figure 1A). The structure of mRNA/V@M NPs was visually characterized by TEM (Figure 1B). To obtain the desired gene CD74 mRNA transfection efficiency, nanoparticles with different N/P ratios were prepared. By agarose gel electrophoresis analysis (Figure S1), it was found that the mRNA was able to fully bind to NPs at a molar ratio of 20. The molar ratio of 20 was used for subsequent experiments. Under fluorescence microscopy, V@M NPs stained with Dil (red) and mRNA labeled with FAM (green) were observed, which could present a clear yellow fluorescence signal (overlap of the lipid coat with the mRNA), indicating successful mRNA loading (Figure 1C). To assess the stability of mRNA/V@M NPs NPs, we performed dynamic light scattering (DLS) measurements in serum (10% FBS in PBS) to simulate the complex vascular environment over 48 h. It can be observed that the hydrodynamic diameters observed in the media do not vary much, which indicates the stability of these NPs (Figure 1D). Dynamic light scattering indicates that the mRNA/V@M NPs have a size of 208 nm (Figure 1E) with a PDI of 0.31. Furthermore, zeta potential measurements showed 35.2 mV for Blank NPs, +6.43 mV for mRNA/V NPs, −6.38 mV for mRNA/V@M NPs, and −25.1 mV for cell membrane (CM). The difference in surface potential may be caused by the negatively charged cell membrane and the loaded mRNA (Figure 1F). The integrity of the membrane coating was further verified by SDS‐PAGE, which showed that the characteristic membrane proteins of CAFs were retained in mRNA/V@M, reflecting their native cellular counterparts (Figure 1H). To assess mRNA stability in nanoparticles, mRNA/V@M nanoparticles were incubated with RNase. Naked mRNA was degraded within 30 min of incubation with ribonuclease. It was observed that mRNA encapsulated in mRNA/V@M NPs did not degrade significantly at 30 min (Figure 1I). Thus, mRNA/V@M nanoparticles can resist RNase‐induced degradation and act as carriers to provide packaging and protection of mRNA.

V‐9302 is an inhibitor targeting the amino acid transporter ASCT2 (SLC1A5) encapsulated in mRNA/V@M NPs. By using UV–Vis spectrophotometer, the drug encapsulation efficiency of V‐9302 was 72%, and the drug loading efficiency was 26.9%. Subsequently, mRNA/V@M under ultrasound stimulation, the drug release reached 74.2%, so the application of ultrasound stimulation (1 W/cm^2^, 60 s, 1 MHz) significantly accelerated the drug release, which was beneficial to the anti‐tumor activity (Figure S2). After the application of ultrasound stimulation (1 W/cm^2^, 60 s, 1 MHz), the drug release was significantly accelerated, which was beneficial to the anti‐tumor activity. The mRNA/V@M nanoparticles containing different concentrations of V‐9302 were prepared, and the results showed that the hemolysis rate gradually increased with the increase of V‐9302 content. The biological safety of the nanoparticles was demonstrated by hemolysis testing (Figure 1G), which showed that the hemolysis rate gradually increased as the content of V‐9302 increased. Through the experiments with different concentration gradients, the IC_50_ of 4T1 cells was 34.89 uM, whereas that of CAF cells was 59.46 uM (Figure S3). In order to improve the safety and tumor cell killing of mRNA nanoparticles, 30 uM V‐9302 was used in the subsequent nanoparticle experiments.

In addition, the ultrasound imaging ability of mRNA/V@M NPs was evaluated. Perfluorohexane (PFH) liquid gas materials can undergo phase transition under ultrasound excitation, transition to a gas state, and form nanobubbles for ultrasound imaging, thereby enhancing ultrasound imaging ability [44]. PFH is a biocompatible phase change material that can be encapsulated in liposome nanoparticles. We then set out to explore the in vitro and in vivo imaging performance of ultrasound‐mediated mRNA/V@M NPs. After therapeutic ultrasound irradiation, the PFH underwent acoustic droplet evaporation, resulting in a uniformly enhanced echo signal in the contrast‐enhanced ultrasound image of mRNA/V@M NPs nanoparticles (Figure 1J). In vivo US imaging revealed an increase in tumor signal intensity on ultrasound images after intravenous injection (Figure 1K). These results demonstrate that the applied US technique can effectively monitor mRNA/V@M NPs imaging in vivo, offering great potential for further exploration of mRNA delivery.

Next, the in vivo biodistribution of mRNA/V@M NP was investigated. In order to simulate the clinical situation of TNBC, co‐cultures of 4T1 and NIH3T3 cells were subcutaneously implanted into BALB/c mice to construct a fibrotic TNBC model rich in extracellular matrix [45]. As shown in Figure 1L, in vivo imaging and quantification were performed after tail vein injection of DiR‐stained mRNA/V@M NPs. The results showed substantial fluorescence accumulation at 4 h in the extracellular matrix‐rich tumors (Figure 1M). Significant fluorescence was also observed in the liver, suggesting that mRNA/V@M NPs may undergo hepatic metabolism. This can be attributed to the fact that lipid nanoparticles are more easily captured by the reticuloendothelial system within the liver.

### 2.2 Ultrasound Enhanced mRNA Transfection and CAF Membrane Mediated Tumor Targeting of NPs@M

**FIGURE 2:**
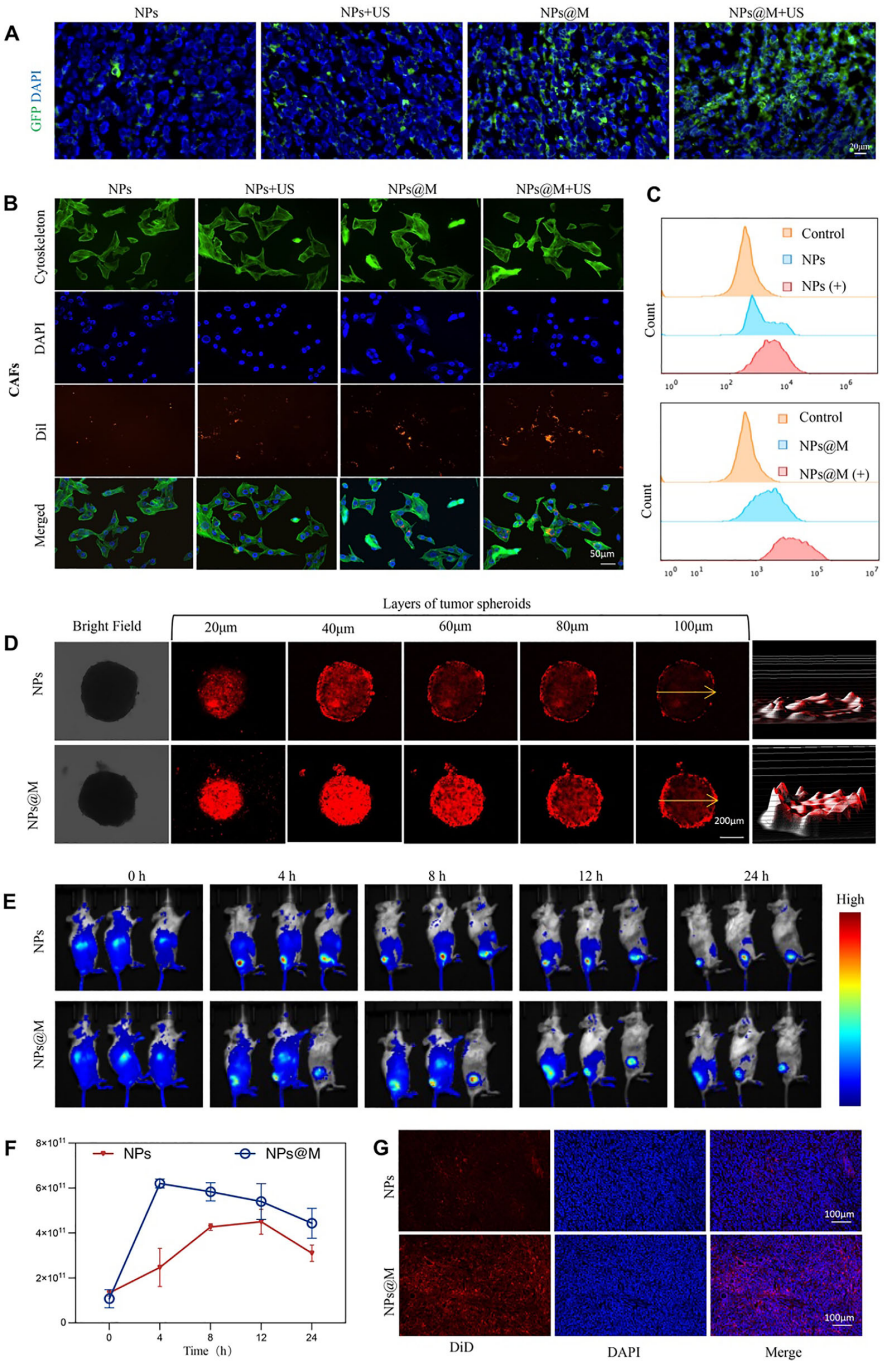
NPs@M delivery of mRNA in vivo and targeting ability of the nanoparticles in vitro and in vivo. (A)  Fluorescence images of the GFP expression in tumor tissues with different treatments. (B) Confocal microscopy visualized nanoparticle internalization in CAFs using triple‐fluorescence staining: nuclei (DAPI, blue), cytoskeletons (Actin‐Tracker Green, green), and nanoparticles (Dil, red). Scale bar, 50 µm. (C) Flow cytometry showing the cell uptake experiment. (D) Targeting ability of NPs and NPs@M in a 3D co‐culture model in different Z‐planes. 4T1 and CAFs were cultured in a 2:1 mixture. Scale bars, 200 µm. (E) Fluorescent distribution of fibrotic TNBC models at different times after intravenous injection of NPs and NPs@M. (F) Fluorescence quantification of tumor tissues. (G) Representative fluorescence intensity of NPs and NPs@M accumulated in tumor sections. Scale bar, 100 µm.

The homologous targeting and camouflage of CAF cell membrane ensure efficient delivery of nanoparticles to the tumor site, and ultrasound irradiation promotes mRNA release into the cytoplasm, which facilitates mRNA transfection [46]. Next, we investigated whether NPs@M+US could efficiently deliver functional mRNA to the cytosol for translation. To investigate this, a fibrotic TNBC model enriched in extracellular matrix was constructed. The mRNA of GFP (GFP mRNA) was loaded into NPs and NPs@M, respectively. After 48 h of treatment with GFP mRNA containing NPs or NPs@M, the translated GFP green fluorescence signal was observed throughout the tumor tissue, and notably, NPs@M combined with US irradiation under commonly used ultrasound mediated delivery conditions (1 W/cm^2^, 60 s, 1 MHz) [47, 48, 49], which balance cavitation assisted nanoparticle penetration and biosafety, resulted in abundant GFP fluorescence throughout the tumor tissue (Figure 2A). Experimental data demonstrated successful mRNA delivery by the nanoparticles, with ultrasound irradiation significantly enhancing translational efficiency, potentially by promoting lysosomal escape [50].

To evaluate nanoparticle internalization and stromal distribution, Dil labeled NPs and NPs@M were examined in CAFs and a fibrotic 3D model. In CAFs, NPs produced only weak red fluorescence, whereas NPs@M showed enhanced internalization, which was further increased by ultrasound irradiation (Figure 2B). This enhancement was further confirmed by flow cytometry, as the proportion of Dil positive cells was higher in the NPs@M + US group than in the NPs@M group, and was also increased in the NPs + US group compared with the NPs group (Figure 2C), indicating that ultrasound effectively promoted nanoparticle uptake, likely through acoustic cavitation mediated membrane permeabilization and PEG lipid shedding. To assess stromal distribution, an ECM rich 3D TNBC spheroid model was established by co‐culturing 4T1 cells with NIH3T3 fibroblasts. NPs@M displayed stronger fluorescence accumulation and deeper penetration within fibrotic spheroids than NPs (Figure 2D). Moreover, free CAF membrane pre‐incubation markedly reduced NPs@M uptake in CAFs and attenuated its penetration into 3D spheroids (Figure S4A,B), indicating that CAF membrane‐mediated homologous recognition may contribute substantially to the improved stromal distribution of NPs@M, in addition to possible physicochemical effects introduced by membrane coating.

Next, the in vivo targeting of NPs@M was investigated. In vivo imaging after intravenous injection of NPs@M showed greater targeting of fibrotic TNBC than NPs (Figure 2E,F). Fluorescence imaging of the extracted tumor tissue sections after the 4 h intravenous injection showed substantial fluorescence aggregates in the extracellular matrix rich tumors (Figure 2G). In conclusion, NPs@M demonstrated excellent ECM targeting in both in vitro and in vivo fibrotic TNBC models.

### 2.3 Reducing Fibroblast Activation Using mRNA/V@M NPs + US

**FIGURE 3:**
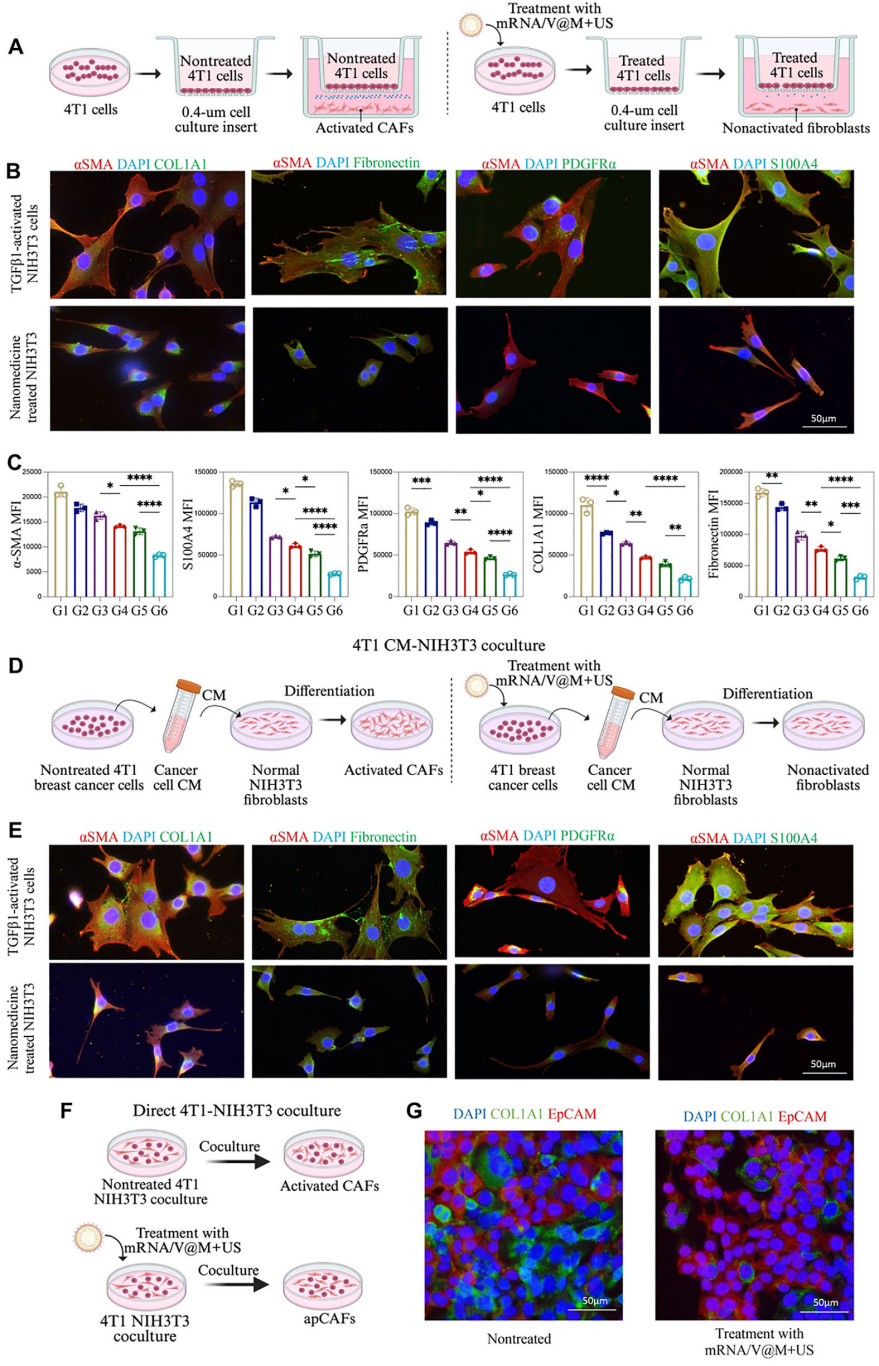
CAFs Reprogramming and ECM Remodeling by mRNA/V@M+US in vitro. (A) The transwell coculture system comprised 4T1 breast cells seeded in 0.4 µm pore upper chambers and NIH/3T3 fibroblasts in lower chambers. Left panel: Co‐culture with untreated 4T1 cells; Right panel: 4T1 cells pre‐treated for 24 h with mRNA/V@M NPs+US (1 MHz, 1 W/cm^2^, 60 s). Schematics created with BioRender. (B) Immunofluorescence analysis of CAF/ECM activation biomarkers in NIH/3T3 fibroblasts following 48 h compartmentalized co‐culture with mRNA/V@M NPs+US‐preconditioned 4T1 carcinoma cells. Scale bars, 50 µm. (C) Flow cytometry quantification of fibroblast activation and ECM remodeling markers in NIH/3T3 fibroblasts following 48 h compartmentalized co‐culture with differentially preconditioned 4T1 carcinoma cells. Group: G1, Control; G2, mRNA NPs; G3, V‐9302 NPs; G4, mRNA/V NPs; G5, mRNA/V@M NPs; G6, mRNA/V@M NPs +US (1 W/cm^2^, 60 s, 1 MHz). ^*^ *p* < 0.05, ^**^ *p* < 0.01, ^***^ *p* < 0.001 and ^****^ *p* < 0.0001 were statistically analyzed by One‐way ANOVA. (D, E) NIH/3T3 fibroblasts exposed for 48 h to conditioned medium from 4T1 cells preconditioned with mRNA/V@M NPs+US (1 MHz, 1 W/cm^2^, 60 s) were subjected to fluorescence microscopic visualization. Scale bars, 50 µm. (F, G) Fluorescence microscopic visualization of NIH/3T3 fibroblasts engaged in direct contact co‐culture with 4T1 carcinoma cells (1:1 ratio) for 24 h following mRNA/V@M NPs+US (1 MHz, 1 W/cm^2^, 60 s). Scale bar, 50 µm. The schematic diagram was created by BioRender.

Cancer cells maintain myCAF activation through paracrine signaling mediators that drive transcriptional and phenotypic reprogramming of fibroblasts. To investigate whether mRNA/V@M NPs + US treatment could interfere with tumor cell induced fibroblast activation, 4T1 breast cancer cells were first treated with different nanoparticle formulations for 24 h and then indirectly co‐cultured with NIH3T3 fibroblasts using a Transwell system, which allowed the transfer of tumor‐secreted factors to fibroblasts without direct cell‐cell contact (Figure 3A). Compared with fibroblasts co‐cultured with untreated 4T1 cells, those co‐cultured with mRNA/V@M NPs + US treated 4T1 cells markedly attenuated fibroblast activation and induced a less contractile fibroblast‐like morphology distinct from activated CAFs. Immunofluorescence staining further showed decreased expression of CAF activation markers, including α‐SMA, S100A4, and PDGFRα, as well as ECM components, including COL1A1 and fibronectin, indicating reduced tumor‐induced fibroblast activation and ECM remodeling (Figure 3B). Consistently, flow cytometric analysis confirmed a coordinated decrease in α‐SMA, COL1A1, S100A4, PDGFRα, and fibronectin expression in fibroblasts co‐cultured with mRNA/V@M NPs + US treated 4T1 cells compared with those co‐cultured with untreated tumor cells (Figure 3C). These results suggest that mRNA/V@M NPs + US treatment suppresses tumor cell driven myCAF activation and attenuates CAF associated ECM remodeling in vitro.

These findings were further validated using two alternative methodologies excluding Transwell systems. The first approach involved NIH3T3 fibroblast exposure to conditioned medium derived from 4T1 cells ‐pretreated with mRNA/V@M NPs+US (Figure 3D). The second paradigm employed direct co‐cultivation of 4T1 cells with NIH3T3 fibroblasts (Figure 3F). The cells were then treated with mRNA/V@M NPs+US (1 W/cm^2^, 60 s, 1 MHz) for 24 h, followed by further co‐culture for 48 h. In both models, mRNA/V@M NPs+US treatment inhibited fibroblast activation while reducing COL1A1 expression (Figure 3E,G). As mentioned earlier, the secretion of stimulating factors and trophic factors in cancer cells stimulates key signaling pathways in CAFs and maintains their activated phenotype. We suggest that mRNA/V@M NPs+US can block the maintenance of the activated CAF phenotypic state by interfering with the secretion of multiple factors by tumor cells. Biomarkers in fibroblasts were downregulated after treatment of cancer cells with mRNA/V@M NPs+US.

### 2.4 Potential of mRNA/V@M to Reprogramming the myCAFs Phenotype

**FIGURE 4:**
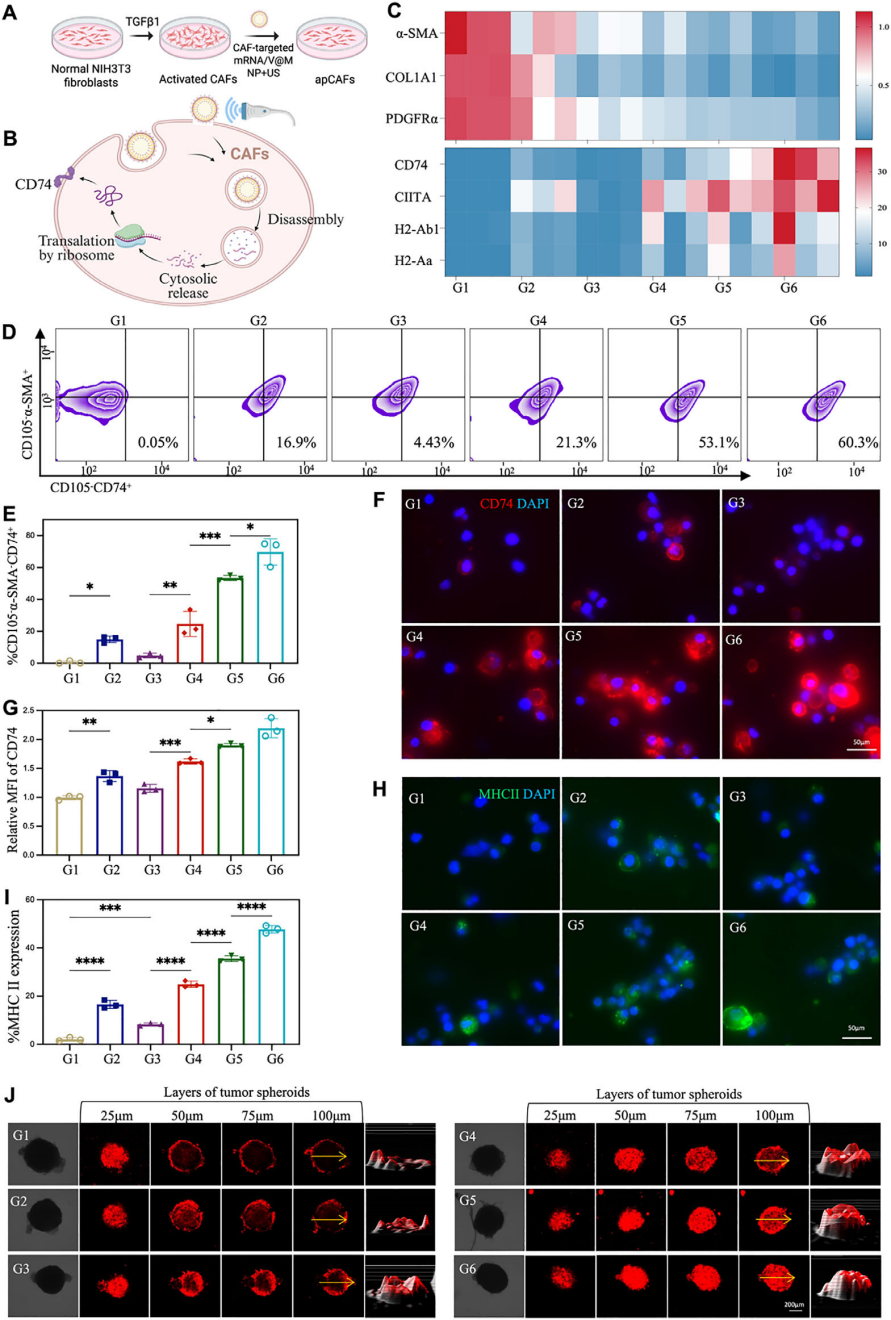
Switching myCAFs into an apCAF phenotype. (A) Schematic shows NIH3T3 fibroblasts preactivated to myCAFs by treatment with TGF‐β1 (10 ng/mL) for 12 h, followed by treatment with mRNA/V@M+US (1 W/cm^2^, 60 s, 1 MHz) (the schematic diagram was created by BioRender). (B) Schematic showing mRNA/V@M nanoparticles targeting CAFs. Nanoparticles enhance CAFs uptake by increasing cell membrane permeability through UTMD, and ribosomes promote mRNA translation to generate CD74 molecules, inducing a shift of the activated myCAFs phenotype to an apCAF phenotype. (C) qPCR analysis of additional CAF subtype related markers after different treatments. Expression levels of myCAF markers (α‐SMA, PDGFR, and COL1A1) and apCAF markers (CD74, CIITA, H2‐Ab1, and H2‐Aa) were evaluated. (D, E) Flow cytometry analysis and statistics of apCAF activation (CD105^−^αSMA^−^CD74^+^) markers after treatment. (F, G) Fluorescence images and statistics of CD74 expression after treatment. (H) Fluorescence pattern of MHC II expression after treatment. (I) Flow cytometry statistics of apCAF activation (MHC II) markers after treatment. (J) Fluorescent images of drug cross sections at different positions and distance‐based photometric quantification after incubating tumor spheroids with different treatments. Group: G1, Control; G2, mRNA NPs; G3, V‐9302 NPs; G4, mRNA/V NPs; G5, mRNA/V@M NPs; G6, mRNA/V@M NPs +US (1 W/cm^2^, 60 s, 1 MHz). ^*^ *p* < 0.05, ^**^ *p* < 0.01, ^***^ *p* < 0.001 and ^****^ *p* < 0.0001 were statistically analyzed by One‐way ANOVA.

According to literature reports, the CD74 gene in the antigen presentation signaling pathway is related to the MHC II signaling pathway, and increased CD74 expression results in a more pronounced MHC II antigen presentation process [20, 29]. We propose that the induced myCAFs can be reprogrammed to the apCAFs phenotype through CD74 mRNA delivery (Figure 4A). Next, we investigated the ability of nanoparticles to reprogram activated myCAFs to an apCAF phenotype (Figure 4B). First, we treated CAFs with different groups in vitro. Given the functional heterogeneity of CAFs, CAF reprogramming was further characterized using an expanded panel of CAF subtype‐associated markers (Figure 4C). The qPCR analysis showed that mRNA/V@M NPs + US markedly decreased the expression of myCAF associated genes, including α‐SMA, PDGFRα, and COL1A1, while increasing apCAF related genes, including CD74, CIITA, H2‐Ab1, and H2‐Aa, suggesting activation of an antigen‐presenting transcriptional program. To assess the feasibility of converting myCAFs to apCAFs, successful conversion of the apCAFs (CD105^−^αSMA^−^CD74^+^) phenotype was identified. There was a significant increase in the expression of apCAFs markers (CD105^−^αSMA^−^CD74^+^) as measured by flow cytometry (Figure 4D,E and Figure S5A). The results proved that mRNA/V@M NP+US successfully reprogrammed the myCAFs phenotype to apCAFs. mRNA/V@M NPs enhanced CAFs uptake by increasing cell membrane permeability through UTMD, promoted ribosomal translation of mRNA to produce CD74 molecules, and promoted the expression of CD74 molecules.

The induction of CD74 and MHC II expression was further validated by multiple complementary assays. Fluorescence imaging and western blot analysis confirmed increased CD74 expression in the mRNA/V@M NPs + US group (Figure 4F,G and Figure S6), while immunofluorescence staining and flow cytometry demonstrated elevated MHC II expression (Figure 4H,I and Figure S5B). To decouple the contribution of ultrasound and the carrier from the therapeutic payload, US alone and empty NPs@M + US were included as additional controls; neither treatment efficiently increased α‐SMA^−^CD74^+^ or MHC II^+^ CAF populations, indicating that CAF reprogramming mainly depended on delivery of the therapeutic CD74 mRNA and V‐9302 payload rather than ultrasound alone (Figure S7). Thus, ultrasound mainly served as a delivery enhancing modality to potentiate the therapeutic efficacy of the nanoplatform. Moreover, the reprogramming effect was also reproduced in primary CAFs isolated from 4T1 tumors, as evidenced by increased α‐SMA^−^CD74^+^ and MHC II^+^ CAF populations, decreased α‐SMA expression, downregulated myCAF related genes, and upregulated apCAF related genes (Figure S8). Collectively, these results demonstrate that mRNA/V@M NPs + US induces a coordinated phenotypic transition from myCAFs toward apCAF cells, with ultrasound primarily serving as a delivery amplifying modality to enhance CAF reprogramming.

It has been reported that the CD74‐MHC II pathway can facilitate immune cells penetration into tumor tissues by disrupting the expression of myCAFs [20], a barrier on the tumor surface. Interestingly, the present study found that successfully reprogrammed apCAFs can reduce the tumor surface barrier, remodel the ECM, and promote immune cell infiltration through the CD74‐MHC II pathway. To evaluate the penetration of lipid nanoparticles into fibrotic triple‐negative breast cancer, a 3D hybrid tumor sphere model was constructed by mixing 4T1 cells with CAFs. As shown in Figure 4J, mRNA/V@M NPs+US enhanced the permeability to the tumor sphere compared to the control group, which was better able to penetrate the ECM barrier and thus penetrate deeper regions inside the tumor sphere. These results indicate that mRNA/V@M NPs+US enhanced intratumoral penetration. This enhanced ability was attributed to several factors. First, mRNA/V@M NPs+ US‐induced CAFs reprogramming and ECM remodeling and breaking of the physical barrier of the tumor spheres. The combination of these factors allows the mRNA/V@M NPs to penetrate to deeper depths. In particular, mRNA/V@M NPs could penetrate deeper after ultrasound stimulation, which was also verified by penetration by fluorescence analysis. Second, mRNA/V@M NPs exhibited a favorable tumor killing effect on 4T1, inhibiting and thus blocking the activation of new CAFs cells. It implies the possibility of deep treatment of fibrotic TNBC by mRNA/V@M NPs+US.

### 2.5 mRNA/V@M NPs Exhibited Potent Antitumor Activity by Reprogramming CAF and Inducing ICD In Vitro

**FIGURE 5:**
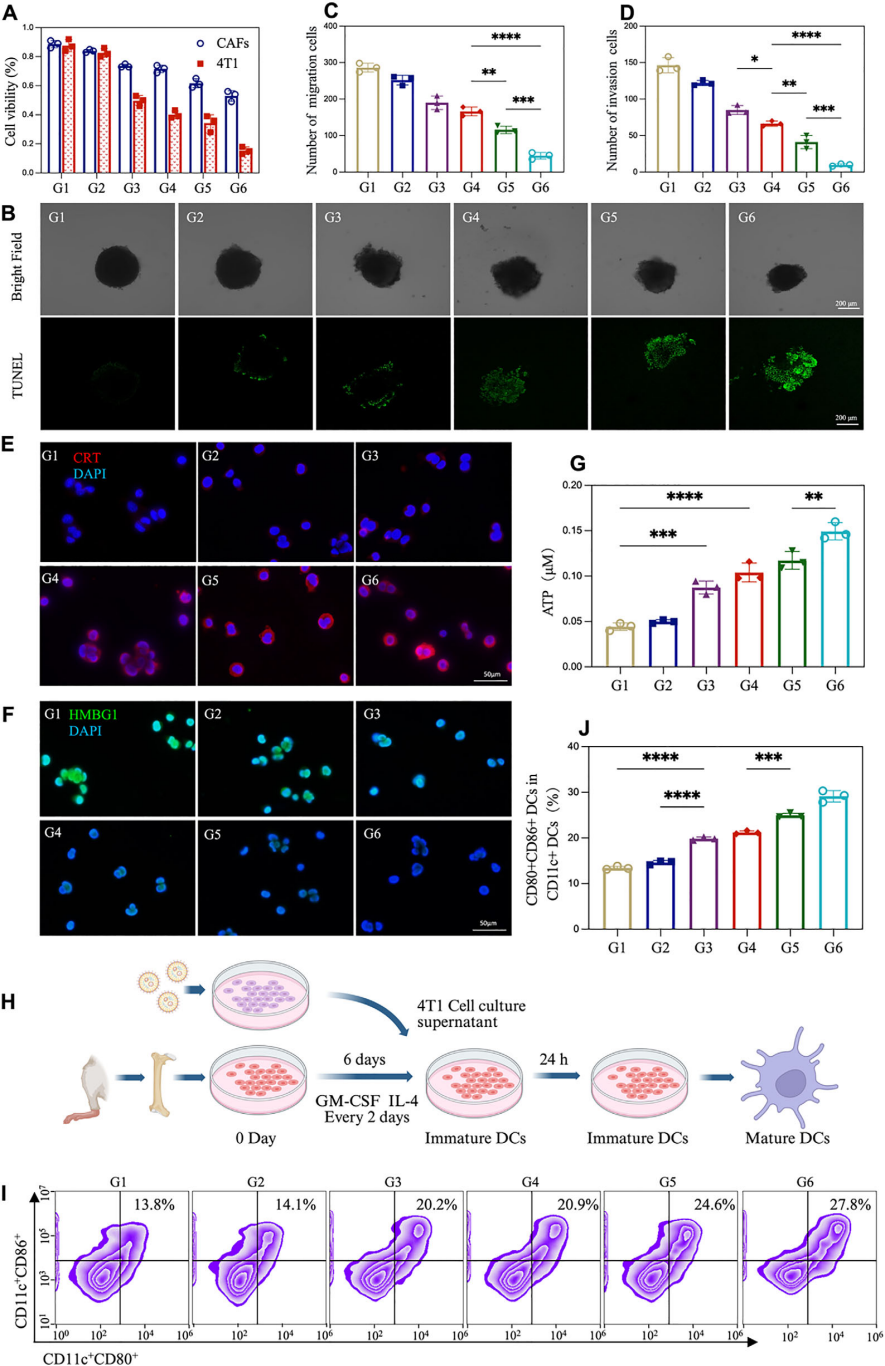
The cellular effects and immune‐related factors of mRNA/V@M NPs on 4T1 were explored. (A) In vitro cytotoxicity in CAFs and 4T1 cells. (B) TUNEL expression in a 3D co‐culture model. Scale bars, 200 µm. (C) In vitro cell migration assay of 4T1 cells. (D) In vitro invasion assay of 4T1 cells. (E) In vitro expression of CRT in 4T1 cells. Scale bars, 50 µm. (F) In vitro expression of HMGB1 in 4T1 cells. Scale bars, 50 µm. (G) Extracellular ATP level after treatment. (H) Illustration of the experimental process of BMDCs maturation. (I,J) Flow cytometry analysis of DCs in vitro, and their quantification (n = 3). Group: G1, Control; G2, mRNA NPs; G3, V‐9302 NPs; G4, mRNA/V NPs; G5, mRNA/V@M NPs; G6, mRNA/V@M NPs +US (1 W/cm^2^, 60 s, 1 MHz). ^*^ *p* < 0.05, ^**^ *p* < 0.01, ^***^ *p* < 0.001 and ^****^ *p* < 0.0001 were statistically analyzed by One‐way ANOVA.

Having established the role of mRNA/V@M NPs+US in CAF reprogramming, the direct antitumor effect of mRNA/V@M NPs+US on 4T1 cells was next assessed in 4T1 cells. As shown, cytotoxicity assays showed (Figure 5A) that mRNA/V@M NPs+US significantly induced cell death in 4T1 cells. Notably, CAF cells exhibited higher cell viability, suggesting that mRNA/V@M NPs+US not only reprogrammed CAF but also enhanced direct tumor suppression. The direct antitumor effect of mRNA/V@M NPs+US on 4T1 cells was next assessed in a co‐culture system of 4T1 cells and CAFs. As shown (Figure 5B), Tunel staining analysis showed that mRNA/V@M NP+US significantly induced cell death in 3D cell spheres in a co‐culture system consisting of 4T1 cells and CAFs. The inhibitory effect of mRNA/V@M NPs+US on the proliferation of 4T1 cells was next assessed in 4T1 cells. The fluorescence plot showed that the positive expression of EdU in the mRNA/V@M NPs+US treatment group was significantly lower than that in the control group, confirming the inhibitory effect of mRNA/V@M NPs+US on proliferation (Figure S9). In addition, the invasion and cell migration assays provided more evidence (Figure S10) that mRNA/V@M NPs+US effectively inhibited tumor cell infiltration and migration and enhanced its potent antitumor activity (Figure 5C,D).

Given the potent antitumor effect of mRNA/V@M NPs+US in vitro, we next explored whether it could enhance ICD‐mediated cell death in 4T1 cells. Mechanistically, V‐9302 mediated glutamine transport blockade disrupts glutamine‐dependent redox homeostasis and promotes oxidative/metabolic stress, which may trigger ICD associated DAMP release [41, 51, 52, 53, 54]. Therefore, in our study, typical ICD biomarkers, including CRT, HMGB1, and ATP, were measured on 4T1 cells to investigate the immune response induced by mRNA/V@M NP+US. With the addition of V‐9302, the immunofluorescence of CRT on the cell membrane was significantly enhanced (Figure 5E), while intracellular HMGB 1 was greatly reduced (Figure 5F). Upon US stimulation, mRNA/V@M NPs treated 4T1 cells exhibited markedly increased CRT exposure and reduced intracellular HMGB1 retention (Figure S11). In parallel, ATP secretion was substantially enhanced in the mRNA/V@M NPs + US group compared with blank tumor cells (Figure 5G). Taken together, the high expression of CRT coupled with the extracellular release of HMGB 1 and ATP confirmed that mRNA/V@M NPs combined with US stimulation potently induced tumor cell ICD and enhances tumor immunogenicity to promote DC maturation.

Subsequently, the potential of mRNA/V@M NPs in combination with US‐mediated anticancer immunity was evaluated by assessing the maturation of dendritic cells (DC). BMDCs cells were incubated with 4T1 cells pretreated with different formulations, and DCmaturation was quantified by measuring the expression of CD80^+^CD86^+^ using flow cytometry analysis (Figure 5H). Consistently, mRNA/V@M NPs + US treatment markedly promoted DC maturation, as evidenced by the increased proportion of CD80^+^CD86^+^ DCs compared with the control group (Figure 5I,J and Figure S12). These results indicate that mRNA/V@M NPs can enhance the ICD effect, and that mRNA/V@M NPs combined with US effectively promote DC maturation, thereby enhancing tumor immunogenicity. Immune related cytokines, including IFN‐β, TNF‐α, and IL‐6, were measured in the supernatant by ELISA (Figure S13). mRNA/V@M NPs+US resulted in a large increase in cytokine concentrations. It is reasonable to speculate that treatment of 4T1 cells with mRNA/V@M NPs + US induces substantial IFN‐β secretion, which promotes DC maturation, enhances MHC I mediated antigen presentation, and subsequently activates antitumor immune responses. Overall, these results indicate that mRNA/V@M NPs+US can potently induce tumor cell ICD to enhance antitumor immune responses through MHC I signaling.

### 2.6 Coordinated Activation of MHC II‐CD4^+^ and MHC I‐CD8^+^ T Cell Axes by CAF Reprogramming and ICD Induction

**FIGURE 6:**
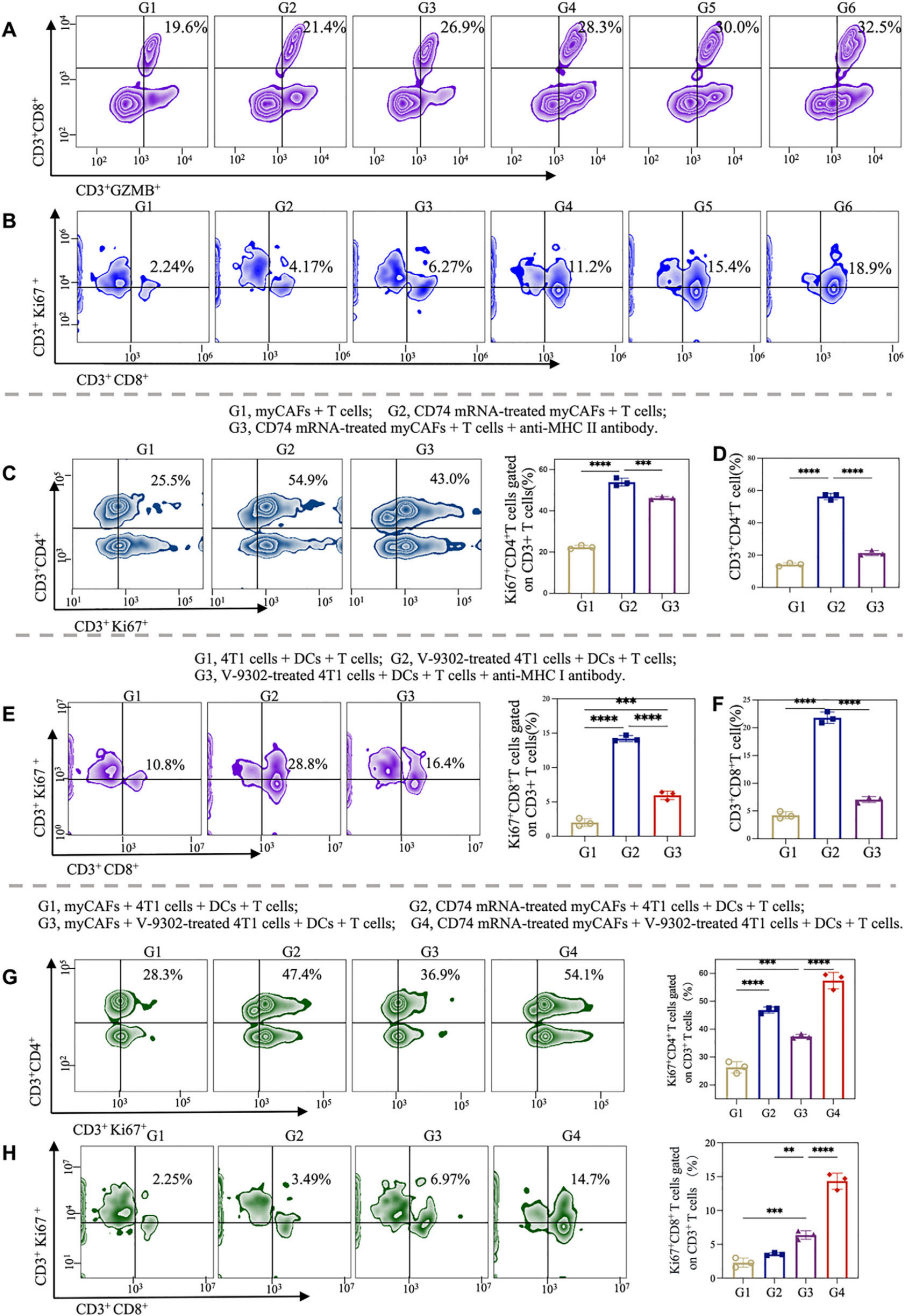
CAF reprogramming and ICD cooperatively enhance T cell activation through dual MHC‐dependent pathways. (A) Representative flow cytometry plots of CD3^+^CD8^+^GZMB^+^ T cells in the integrated co‐culture system consisting of myCAFs, 4T1 tumor cells, DCs, and T cells. Quantitative analysis is shown in Figure S14B. (B) Representative flow cytometry plots of CD3^+^CD8^+^Ki67^+^ T cells in the integrated myCAF/4T1/DC/T cell co‐culture system. Quantitative analysis is shown in Figure S14C. Group: G1, Control; G2, mRNA NPs; G3, V‐9302 NPs; G4, mRNA/V NPs; G5, mRNA/V@M NPs; G6, mRNA/V@M+US (1 W/cm2, 60 s, 1 MHz). (C) Representative flow cytometry plots and quantitative analysis of CD3^+^CD4^+^Ki67^+^ T cells in the MHC II blocking assay using the CAF/T cell co‐culture system. (D) Quantitative analysis of CD3^+^CD4^+^ T cells in the MHC II blocking assay. (E) Representative flow cytometry plots and quantitative analysis of CD3^+^CD8^+^Ki67^+^ T cells in the MHC I blocking assay using the 4T1/DC/T cell co‐culture system. (F) Quantitative analysis of CD3^+^CD8^+^ T cells in the MHC I blocking assay. (G) Representative flow cytometry plots and quantitative analysis of CD3^+^CD4^+^Ki67^+^ T cells in the integrated co‐culture system consisting of myCAFs, 4T1 tumor cells, DCs, and T cells. (H) Representative flow cytometry plots and quantitative analysis of CD3^+^CD8^+^Ki67^+^ T cells in the integrated co‐culture system consisting of myCAFs, 4T1 tumor cells, DCs, and T cells. Data are presented as mean ± SD, n = 3. Statistical significance was determined by one‐way ANOVA. ^*^ *p* < 0.05, ^**^ *p* < 0.01, ^***^ *p* < 0.001, and ^****^ *p* < 0.0001.

Given that ICD mediated antitumor immunity depends on downstream T cell activation, we next investigated whether V‐9302 induced ICD and CD74 mRNA‐mediated CAF reprogramming could cooperatively activate T cells through the MHC I‐CD8^+^ and MHC II‐CD4^+^ axes. To further determine whether this ICD driven immune activation could enhance downstream T cell responses, we established an integrated co‐culture system consisting of myCAFs, 4T1 tumor cells, DCs, and T cells. Flow cytometric analysis showed that mRNA/V@M NPs + US markedly increased CD3^+^CD8^+^ T cells (Figure S14A), as well as CD3^+^CD8^+^GZMB^+^ (Figure 6A and Figures S14B and S15A) and CD3^+^CD8^+^Ki67^+^ T cells (Figure 6B and Figures S14C and S15B), indicating enhanced CD8^+^ T cell expansion, cytotoxic effector function, and proliferative activation. These findings demonstrate that V‐9302 induced ICD promotes DC mediated CD8^+^ T cell activation and cooperates with CD74 mRNA mediated CAF reprogramming to amplify adaptive antitumor immunity.

To dissect the respective contributions of CD74mRNA mediated CAF reprogramming and V‐9302 induced ICD to T cell activation, we further performed pathway specific co‐culture and blocking assays. For the MHC II‐CD4^+^ T cell axis, a myCAF/T cell co‐culture system was established. Compared with untreated myCAFs, CD74 mRNA treated myCAFs markedly increased the proportions of CD4^+^ T cells and Ki67^+^CD4^+^ T cells (Figure 6C,D). Notably, MHC II blockade substantially attenuated these effects, confirming that CD74 mRNA induced apCAF reprogramming activates CD4^+^ T cells primarily through an MHC II dependent pathway.

For the MHC I‐CD8^+^ T cell axis, a 4T1/DC/T cell co‐culture system was used to evaluate V‐9302 mediated tumor immunogenicity. V‐9302 treated 4T1 cells significantly increased the proportions of CD8^+^ T cells and Ki67^+^CD8^+^ T cells, whereas MHC I blockade markedly reduced CD8^+^ T cell activation and proliferation. These results indicate that V‐9302 induced ICD promotes downstream CD8^+^ T cell activation through MHC I dependent antigen presentation (Figure 6E,F).

We next evaluated whether these two immune activation routes could cooperate in an integrated co‐culture system containing tumor, stromal, and immune components. A co‐culture system consisting of myCAFs, 4T1 cells, DCs, and T cells was established to compare the effects of CD74 mRNA treated myCAFs, V‐9302 treated 4T1 cells, and their combination. The combined treatment induced the most pronounced increases in CD4^+^ T cells (Figure S14D,G), Ki67^+^CD4^+^ T cells (Figure 6G and Figure S15C), CD8^+^ T cells (Figure S14E,F), and Ki67^+^CD8^+^ T cells (Figure 6H), indicating coordinated activation of both CD4^+^ T cells and CD8^+^ T cells responses. Together, these findings demonstrate that CD74 mRNA mediated CAF reprogramming and V‐9302 induced ICD cooperatively engage the MHC II‐CD4^+^ and MHC I‐CD8^+^ T cell axes, thereby establishing a dual antigen presentation circuit that amplifies adaptive antitumor immunity beyond a simple additive effect.

### 2.7 Antitumor Effect of mRNA/V@M NPs on Fibrotic TNBC Model

**FIGURE 7:**
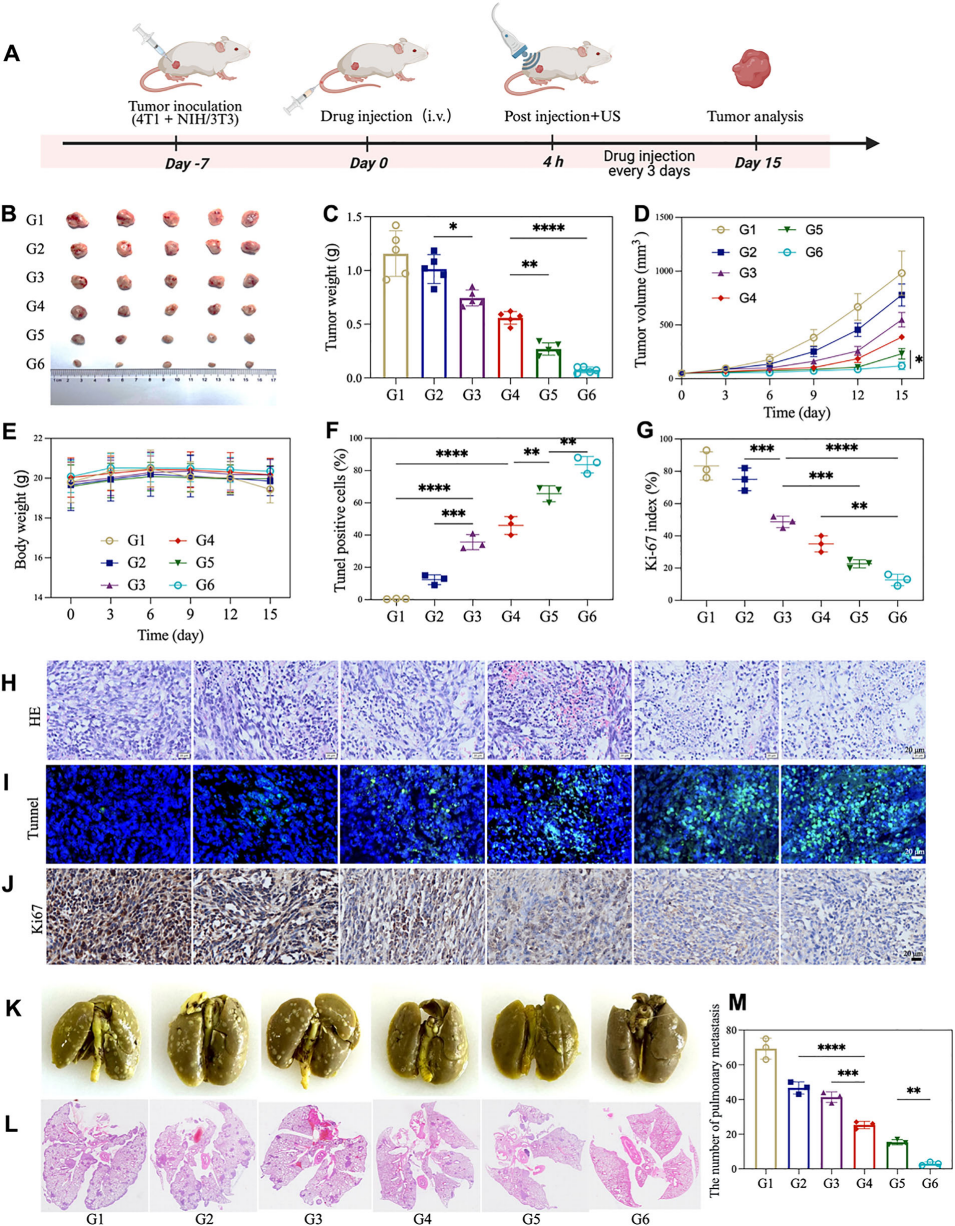
Inhibition of tumor growth and metastasis by mRNA/V@M+US. (A) Schematic of the treatment in the fibrotic TNBC tumor model (the schematic diagram was created by BioRender). (B) Images of tumors and the weight (C) in each group after different treatments. (D) Tumor growth curves of each group. (E) Weight changes of mice during treatments. (H) H&E staining of mouse tumor sections after different treatments. Scale bars, 20 µm. (I) TUNEL staining of the tumor sections and quantitative analysis (F) of Tunel positive 4T1 cells of the tumor sections (n =3). Scale bars, 20 µm. (J) Ki67 immunohistochemistry staining and Ki67 index analysis (G) of the tumor sections (n =3). Scale bars, 20 µm. (K) Representative images of metastasis of lungs. (L) H&E stained images of mouse lung sections after various treatments. (M) Statistics of metastasis of lungs. Group: G1, Control; G2, mRNA NPs; G3, V‐9302 NPs; G4, mRNA/V NPs; G5, mRNA/V@M NPs; G6, mRNA/V@M NPs +US (1 W/cm^2^, 60 s, 1 MHz). ^*^ *p* < 0.05, ^**^ *p* < 0.01, ^***^ *p* < 0.001 and ^****^ *p* < 0.0001 were statistically analyzed by One‐way ANOVA.

Previous experiments have shown that the combination of mRNA/V@M NPs+US enhances anti‐tumor ability by reprogramming CAF to enhance MHC II as well as inducing enhanced MHC I pathway. Therefore, we investigated the antitumor effect of altering the apCAF phenotype in vivo. To mimic the clinical situation of fibrotic TNBC, a mixture of 4T1 and NIH3T3 cellswere co‐implanted in the right dorsal region of mice at a ratio of 2:1 (Figure 7A). First, the tumor‐bearing mice were treated with different groups of drugs by tail vein injection. When the tumor volume of BALB/c mice reached ≈50 mm^3^, the mice were randomly divided into different groups (n = 5 per group). Then, the corresponding materials were injected intravenously every 3 days, and ultrasound (1.0 W/cm^2^, 60 s, 1.0 MHz) was given 4 h after injection based on the above results. However, there was a significant difference in tumor volume between the groups as compared with the control group (Figure 7B). The mRNA/V@M NPs showed a significant tumor inhibitory effect, while the mRNA/V@M NPs+US showed a better anti‐tumor effect. These results were further validated by changes in tumor weight after treatment (Figure 7C). In contrast, mRNA/V@M NPs treatment substantially suppressed tumor growth, and this antitumor effect was further enhanced by US irradiation, as reflected by the markedly reduced tumor weight compared with the control group. The untreated control group exhibited aggressive tumor expansion, while single‐agent mRNA NPs or V‐9302 NPs mediated only modest growth inhibition. In striking contrast, combinatorial nanoplatforms co‐encapsulating mRNA and V‐9302 elicited potent tumor suppression (Figure 7D). It is encouraging that ultrasound‐triggered mRNA/V@M NPs elicited complete tumor stasis, which may be due to the transformation of myCAF into apCAFs with immune activation function to enhance the MHC II pathway, combined with the effective and highly activated ICD effect of MHC I signaling, and the immune cell infiltration caused by the reprogramming of CAF phenotype. The increased tumor suppression efficiency of mRNA/V@M NPs compared to the mRNA/V NPs group suggests that the addition of biological targeting could facilitate V‐9302 crossing the CAF barrier and improve the therapeutic efficacy. mRNA/V@M nanoparticles demonstrated favorable biocompatibility profiles, evidenced by maintained body weight stability throughout the dosing period and unaltered histoarchitecture in H&E‐stained major organ sections (Figure 7E and Figure S16).

In addition, H&E (Figure 7H) and TUNEL (Figure 7I) staining of tumor sections indicated that the mRNA/V@M NPs+US group, which expressed the most apCAF phenotype, increased tumor cell death(Figure 7F). In addition, Ki67 was also inhibited in the tumors (Figure 7J,G), confirming the tumor proliferation inhibition of mRNA/V@M NPs+US.

Given the high metastatic potential of fibrotic TNBC, inhibition of lung metastasis is also a key factor in evaluating the efficacy of treatment. To assess the antimetastatic effect of nanoparticles, a lung metastasis model was established, and lungs from each group were collected for evaluation after treatment (Figure 7K). After treatment, the number of lung metastases was reduced in the treatment group compared with the control group, especially in the mRNA/V@M NPs+US group, which had very few metastatic lesions compared to the control group (Figure 7M). H&E staining of lung tissue further confirmed (Figure 7L) that engineered apCAF phenotypic alteration in conjunction with tumor suppression could effectively inhibit lung metastasis.

### 2.8 Modulation of the Immunosuppressive TME and ECM Remodeling by mRNA/V@M+US

**FIGURE 8:**
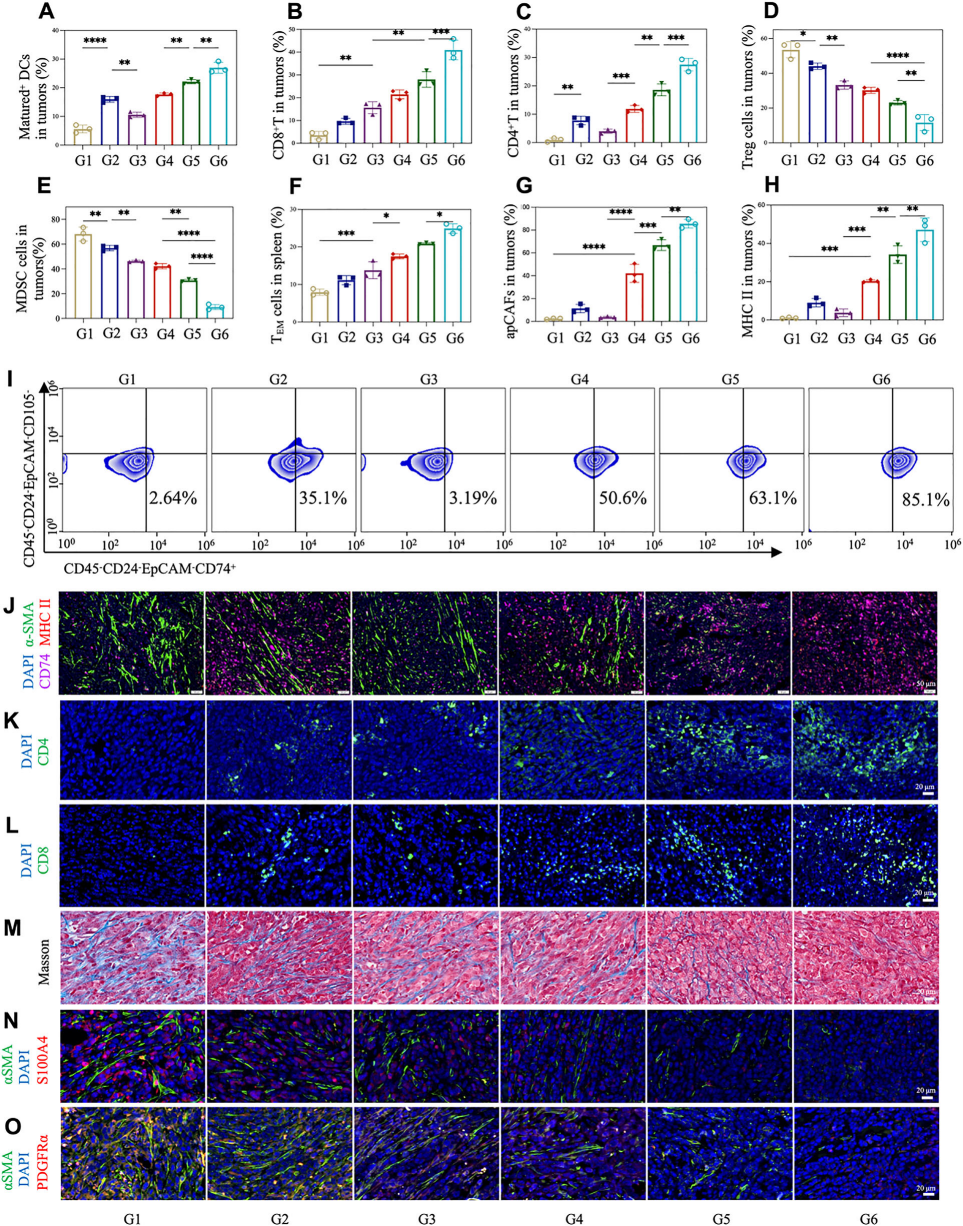
Immune cell analysis in primary tumors after different therapy. (A) Flow cytometric quantification of matured DCs infiltration in tumor tissues marked with CD11c^+^CD86^+^CD80^+^. n =3. (B) Flow cytometric quantification of cytotoxic T cells (CTLs) in tumors marked with CD3^+^CD8^+^. n =3. (C) Flow cytometric quantification of CD4^+^T cells in tumors marked with CD3^+^CD4^+^. n =3. (D) Flow cytometric quantification of regulatory T cells (Tregs) in tumors marked with CD4^+^CD25^+^FOXP3^+^. n =3. (E) Flow cytometric quantification of MDSC cells in tumors marked with CD11b^+^Gr‐1^+^. n =3. (F) Flow cytometric quantification of T_EM_ (CD3^+^CD8^+^CD44^+^CD62L^−^) in the spleen. n =3. (G) Flow cytometric quantification of antigen‐presenting CAF (apCAFs) in tumors marked with CD45^−^CD24^−^EpCAM^−^CD105^−^CD74^+^. n=3. (H) Flow cytometric quantification of MHC‐II^+^ cells in tumors. n =3. (I) Immunophenotyping analysis showing abundance of apCAFs (CD45^−^CD24^−^EpCAM^−^CD105^−^CD74^+^). (J) Representative multicolor immunofluorescence staining of tumor tissues with DAPI, α‐SMA, CD74, and MHC II, showing in situ CAF phenotypic remodeling after different treatments. Scale bars, 50 µm. (K) immunofluorescence staining of CD4^+^ T cells. Scale bars, 20 µm. (L) immunofluorescence staining of CD8^+^ T cells. Scale bars, 20 µm. (M) Representative images of Masson trichrome staining. (N, O) Immunofluorescence imaging of myCAF markers in tumor sections after different treatments. Scale bars, 20 µm. Group: G1, Control; G2, mRNA NPs; G3, V‐9302 NPs; G4, mRNA/V NPs; G5, mRNA/V@M NPs; G6, mRNA/V@M NPs+US (1 W/cm^2^, 60 s, 1 MHz). ^*^ *p* < 0.05, ^**^ *p* < 0.01, ^***^ *p* < 0.001 and ^****^ *p* < 0.0001 were statistically analyzed by One‐way ANOVA.

To verify the regulatory effect of mRNA/V@M NPs+US on immunosuppressive TME and stroma, we constructed a fibrotic TNBC model rich in extracellular matrix and treated it in groups in vivo. To assess MHC I pathway activation, tumor‐infiltrating mature DCs were analyzed by flow cytometry. The treatment markedly increased the proportion of CD11c^+^CD86^+^CD80^+^ DCs compared with the blank group, indicating enhanced intratumoral DC maturation (Figure 8A and Figure S17A). The increased activation and antigen presentation capacity of DCs in tumors suggests an effective systemic antitumor response. We next assessed intratumoral T cell infiltration. Flow cytometric analysis showed that treatment markedly increased the proportions of both CD3^+^CD8^+^ T cells (Figure 8B and Figure S17B) and CD3^+^CD4^+^ T cells (Figure 8C and Figure S17C) compared with the blank group, indicating enhanced adaptive immune activation within the tumor microenvironment. The enrichment of T cells within the tumor, as well as ICD mediated immunogenic death, promotes robust tumor recognition and eradication. Importantly, CAF reprogramming relieved the immunosuppressive tumor microenvironment [55], leading to marked reductions in regulatory T cells (Tregs) (Figure 8D and Figure S18A) and myeloid‐derived suppressor cells (MDSCs) (Figure 8E and Figure S18B) compared with the blank group.

Effector memory T cells (TEM) (CD44^+^CD62L^−^) play a crucial role in preventing tumor recurrence [56]. Consistently, splenic immune profiling revealed a marked increase in TEM after mRNA/V@M NPs + US treatment, indicating enhanced systemic immune memory formation (Figure 8F and Figure S18C). IFN‐γ and TNF‐α are the major cytokines that perform the antitumor function of CD8^+^ T cells. When the mice in the mRNA/V@M NPs+US group had a significant increase IFN‐γ and TNF‐α concentrations compared with the control group (Figure S19), the mRNA/V@M NPs+US treatment could effectively induce an immune memory effect, enhance systemic anti‐tumor immunity, and prevent tumor recurrence.

We next examined whether CAF phenotypic remodeling occurred in vivo. Multicolor immunofluorescence staining of tumor tissues showed decreased α‐SMA expression and increased CD74/MHC II signals after mRNA/V@M NPs + US treatment, indicating in situ remodeling of myCAFs toward an antigen presenting CAF phenotype (Figure 8J). Consistently, in vivo flow cytometry revealed increased MHC II^+^ cells (Figure 8H and Figure S20A) and enriched apCAFs in tumors (Figure 8G), defined as CD45^−^CD24^−^EpCAM^−^CD105^−^CD74^+^ cells (Figure 8I and Figure S20B). Under the selected ultrasound condition (1 W/cm^2^, 60 s, 1 MHz), mRNA/V@M NPs + US also enhanced CD4^+^ T cell (Figure 8K) and CD8^+^ T cell infiltration (Figure 8L), supporting the suitability of this ultrasound assisted delivery strategy for CAF reprogramming and immune activation in the 4T1 tumor model. Together with the ECM related changes described above, these results establish a mechanistic link among CAF reprogramming, stromal remodeling, and enhanced T cell immunity, and demonstrate the successful induction of an antigen presenting CAF state characterized by elevated CD74 and MHC II expression.

It has been reported that the CD74‐MHC II pathway can promote immune cell penetration into the tumor interior by disrupting the expression of tumor‐associated fibroblasts (CAFs) [20]. CAFs, a barrier on the tumor surface, are currently the focus of research, so we evaluated the tumor microenvironment. As shown, extracellular matrix deposition profiles were histologically assessed via Masson's trichrome staining (Figure 8M), and immunofluorescence staining with activation markers for CAF (αSMA, S100A4, and PDGFR α) (Figure 8N,O) and ECM markers (COL1A1 and Fibronectin) (Figure S21) confirmed tumor ECM remodeling in vivo. In the control group, a large amount of collagen was found to be deposited in the intercellular matrix, whereas very little collagen was present in the mRNA/V@M NPs+US group. All fibroblast activation and expression of ECM markers were lower in tumor tissue samples from the treatment group (mRNA/V@M NPs+US) than in the control group. It was confirmed that mRNA/V@M NPs+US regulated stromal cells after reprogramming CAFs in vivo, reduced extracellular matrix deposition, such as collagen, and increased immune cell infiltration and viability. Taken together, the above results clearly point to a synergistic effect of mRNA/V@M NPs and US activation to break the physical barrier of TNBC by reprogramming CAF and remodeling ECM, significantly increasing CD8^+^ cell infiltration and remodeling the TME to achieve a robust immunotherapy effect against breast cancer.

## 3 Conclusions

In summary, we have proposed an anti‐tumor strategy that combines CAFs reprogramming with ICD and constructed a spatio‐temporal controllable targeted drug system to inhibit fibrotic TNBC. We reprogrammed the immunosuppressed myCAF into apCAFs with immune activation functions, which then enhanced antigen presentation and activated CD4^+^T cells via the CD74‐MHC II pathway. V‐9302 promotes the activation of ICD effects, thereby overcoming the limitation of MHC I insufficient immunity and significantly enhancing the effect of immunotherapy to achieve complementary synergy. In conclusion, the combination of reprogramming apCAFs and ICD effect, by significantly increasing CD8^+^ cell infiltration, remodeling the TME, and MHC I and MHC II synergistically to achieve a robust immunotherapy effect against breast cancer.

## 4 Materials and Methods

### 4.1 Extraction of Tumor Cell Membranes

In this study, CAF membranes were extracted according to the membrane protein extraction kit instructions. CAF cells were cultured to approximately 10^9^ cell counts, and then cell membrane proteins were extracted using the Membrane Protein Extraction Kit (Beyotime, China). For the cultured cells, the freeze‐thaw method was used to break the cells. The cells were freeze‐thawed twice in liquid nitrogen and at room temperature sequentially and repeatedly. The precipitate was collected, and the cell membrane fragments were resuspended in PBS. Centrifugation was performed at 700 g for 10 min at 4°C, and the supernatant was carefully collected into a new centrifuge tube. Centrifugation was performed at 14 000 g for 30 min at 4°C to precipitate the cell membrane fragments. The precipitate was collected, and the cell membrane fragments were resuspended in PBS. The resuspended cell membrane fragments were then stored at −20°C for further analysis and experimentation.

### 4.2 Preparation of mRNA/V@M NP

The NPs were prepared using the using the methods reported in previous studies [57, 58]. Briefly, 1,2‐Dipalmitoyl‐sn‐glycero‐3‐phosphocholine (DPPC), 3‐[N‐(N',N'‐dimethyl‐lamino‐ethane)‐carbamoyl] cholesterol (DC‐CHOL) and 1,2‐distearoyl‐sn‐glycerol‐3‐phosphoethanolamine‐N‐[maleimide (polyethylene glycol)] (DSPE‐PEG2000), purchased from Ruixibio(China), were weighed and dissolved in 5 mL chloroform at a molar ratio of 5 mg (6.8 µmol): 2 mg (0.67 µmol): 1 mg (2.59 µmol), and then vortexed and evaporated to remove the residual solvents. The mixed film was obtained through the culture‐extrusion method [59]. In summary, in an ice bath, the CAF membrane protein and lipid mixture were mixed at a 1:1 mass ratio (calculated based on protein dose, where the mass of macrophage membrane protein added was equivalent to the mass of cholesterol added), and then subjected to 5 min of ultrasonic treatment. Then, they were extruded 11 times through 0.4 micrometer and 0.2 micrometer polycarbonate porous membranes to promote membrane fusion. The harvested hybrid membranes were stored in PBS at 4°C. Then, they were ultrasonically mixed at 4 degrees for 5 min, and 200 µL of PFH and V‐9302 (5 mg) was added. The pulse mode was set to 5 s on and 5 s off to ensure the uniform dispersion of the drug in the polymer matrix. Subsequently, the emulsion was stirred in a dark environment at 4°C for 4 h. Then, the formulation was centrifuged at 15 000 revolutions per minute for 30 min to purify it, and then washed with water to remove any unbound or excess materials. The emulsion was incubated with mRNA for 30 min at 4°C. It was then washed with water to remove any unbound or excess material.

### 4.3 Characterization of mRNA/V@M NP

The particle size and zeta potential of the NPs were determined by dynamic light scattering (DLS, Zetasizer nano 2S, Malvern). The morphology of the nanoparticles was observed using transmission electron microscopy (TEM, JEM1400PLUS, Japan). The stability of the physiological solution was investigated by dynamic light scattering (DLS). mRNA/V@M NPs were dispersed into PBS containing 10% fetal bovine serum (FBS) to monitor temporal changes in nanoparticle size. Subsequently, drug encapsulation, loading, and drug release of V‐9302 were tested by UV–vis spectroscopy analysis. In addition, to investigate the encapsulation efficiency of mRNA by different nanoparticles, a gel blocking assay was performed. The mRNA was mixed with nanoparticles in different volume ratios and centrifuged, and the upper liquid layer was aspirated for the gel‐blocking test. Briefly, the samples (20 µL) were mixed with 4 µL of 6 × buffer and electrophoresed on a 1% agarose gel. Electrophoresis was performed at 80 volts for 3 min followed by 10 min at 120 volts, and the resulting gel was photographed under UV light. As a control, free mRNA was used.

### 4.4 Cell Lines

The 4T1 cells and NIH/3T3 cells were purchased from Procell system (Wuhan, China). These cells were cultured in DMEM medium, with 10% fetal bovine serum and 1% penicillin‐streptomycin solution added to the medium. They were cultured in a 37°C constant temperature incubator in a humidified environment containing 5% carbon dioxide. The NIH/3T3 embryonic fibroblasts were treated with 10 nanograms per milliliter of TGF‐β1 for 12 h to transform them into a cancer‐associated fibroblast model.

### 4.5 Cellular Model Construction and Drug Penetration Studies of Tumor Spheroids

To simulate drug conditions within solid tumors, ECM rich 3D tumor spheroids were created. Briefly, 4T1 cells and NIH/3T3 cells were mixed and inoculated in a 2:1 ratio in low adsorption 96‐well plates, which were then centrifuged at room temperature, 250 RCF, 2 min The plates were incubated at 37°C for 7 days, with moderate changes occurring halfway through the incubation period. This process promoted the generation of tumor cell spheroids. The plates were then treated with PBS, mRNA NPs, V‐9302 NPs, mRNA/V NPs, mRNA/V@M NPs, and mRNA/V@M+US (1 W/cm^2^, 60 s, 1 MHz) for 24 h. US irradiation (acoustic intensity: 1 W/cm^2^, frequency: 1 MHz, duty cycle: 50%, duration: 60 s) was applied. After washing with PBS, the spheres were transferred to a confocal dish and observed under Super‐resolution spinning‐disk confocal system (Olympus SpinSR10).

### 4.6 Construction of Animal Tumor Models

All animal experiments were approved by the Animal Care and the Animal Committee at the Qilu Hospital of Shandong University (Approval No. DWLL‐202500419). All animal experiments were approved by the Institutional Animal Care and the Animal Committee at the Qilu Hospital of Shandong University. Female BALB/c mice aged 4 to 6 weeks were purchased from the Experimental Animal Center of Shandong University Qilu Hospital (Shandong, China) and were raised under standard breeding conditions. To construct a fibrotic TNBC model, 4T1 and 3T3 cells were implanted into the right dorsal region of mice at a ratio of 2:1. An equal number of 4T1 cells were implanted into the subcutaneous region of the right dorsum to construct the normal TNBC model. Unless indicated, the tumor model in this study defaulted to the fibrotic BRCA model. Seven days after tumor implantation, mice were injected intravenously every 3 days with the appropriate treatment (Control, mRNA NPs, V‐9302 NPs, mRNA/V NPs, mRNA/V@M NPs, mRNA/V@M+US (1 W/cm^2^, 60 s, 1 MHz)). The V‐9302 equivalent dose of 25 mg/kg was selected based on previous studies using comparable V‐9302 doses for tumor metabolic intervention and immune activation [51, 52, 53, 54]. Body weight and tumor volume were measured every 3 days. On day 15, mice were sacrificed, and their tumors were collected, photographed, and weighed. And their tumors were collected for flow analysis, H&E staining, and IHC.

### 4.7 Indirect Co‐Culture of Cancer Cells and Fibroblasts

Transwell co‐culture, 4T1 cancer cells were cultured in Petri dishes and treated for 24 h (mRNA/V@M NPs + US (1.0 W/cm2, 60 s,1.0 MHz)). The treated cells were cultured in the upper wells of the chambers (pore size of 0.4 µm), and then the cell culture chambers were added to a 6‐well plate. NIH/3T3 cells were cultured in the bottom wells and grown to 50% cell density, and then grown in serum‐free DMEM for 24 h to inhibit growth. The Transwell co‐culture system was continued for 24 h to stimulate the fibroblasts to be stimulated by stimulatory factors secreted by the cancer cells, along with amino acids. Fibroblasts were then stained for immunofluorescence imaging and flow cytometry. Antibodies were mouse anti‐αSMA, rabbit anti‐COL1A1, rabbit anti‐α‐S100A4, rabbit anti‐Fibronectin, and rabbit anti‐PDGFR.

### 4.8 Conditioned Medium (CM) Co‐Cultured

To generate conditioned medium for cancer cells, 4T1 cancer cells at 70%–80% cell density were treated for 24 h (mRNA/V@M NPs + US (1.0 W/cm2, 60 s,1.0 MHz)). The culture medium was collected for centrifugation (5 min,1200 rpm/min), and then the supernatant was mixed with cell culture solution (50:50) for stimulation of NIH/3T3 cells. NIH/3T3 cells were cultured in 6‐well plates and grown to 50% cell density, then growth was suspended for 24 h. Subsequently, fibroblasts were co‐cultured with cancer cell culture medium for 24 h. Afterwards, immunofluorescence staining of fibroblasts was performed for imaging. Antibodies were anti‐mouse αSMA, anti‐rabbit COL1A1, anti‐rabbit α‐S100A4, anti‐rabbit Fibronectin, and anti‐rabbit PDGFR.

### 4.9 Direct Co‐Culture of Cancer Cells and Fibroblasts

4T1 cancer cells were co‐cultured with NIH/3T3 cells at a 1:1 ratio (20 000 cells each) in 12 well plates in DMEM medium containing 10% fetal bovine serum. When the cell density reached 70%–80%, the cell co‐cultures were treated with mRNA/V@M NPs + US (1.0 W/cm^2^, 60 s, 1.0 MHz) for 24 h. Finally, the cells were immunofluorescently stained for imaging. Antibodies were anti‐rabbit COL1A1 and anti‐mouse EpCAM.

### 4.10 CAF Phenotype Switching In Vitro and In Mice Model

NIH/3T3 cells were seeded in 6‐well plates at a density of 1 × 10^6^ cells per well and stimulated with the addition of TGF‐β1 for 12 h to convert to CAFs. Then grouped for treatment for 24 h. The treated cells were then collected for membrane‐breaking, staining, and fixation. The percentage of CAFs (α‐SMA^−^CD105^−^CD74^+^) and MHCII were analyzed by FACS.

Collected treated tumors were made into single‐cell suspensions and subsequently stained using a combination of fluorescent labeled antibodies to identify the percentage of CAFs (CD45^−^CD24^−^EpCAM^−^CD105^−^CD74^+^) and MHCII in mouse tumors. After staining, cells were fixed with 4% PFA and analyzed by FACS (Beckman Coulter, Gallios). Data were subsequently analyzed on FlowJo. Collected tumors were dehydrated and fixed, then embedded and sectioned. Sections were closed, permeabilized, and incubated with antibodies. Antibodies were anti‐mouse αSMA, anti‐rabbit COL1A1, anti‐rabbit α‐S100A4, anti‐rabbit Fibronectin, and anti‐rabbit PDGFR. Secondary antibodies were incubated. Cell nuclei were restained with DAPI. The cells were then visualized by fluorescence microscopy.

### 4.11 Immunofluorescence Analysis

NIH/3T3 cells were seeded in 24‐well plates at a density of 1 × 10^5^ cells per well and stimulated with the addition of TGF‐β1 for 12 h to convert to CAFs. Expression of specific proteins was assessed after group treatment for 24 h. Cells were fixed with 4% para‐formaldehyde and closed with goat serum and permeabilized with Triton X‐100. Cells were then incubated overnight at 4 degrees with target protein antibodies. Nuclear staining was performed using DAPI to visualize nuclei. Fluorescence Inverted Microscope (Olympus IX73) was used to examine the expression and distribution of α‐SMA, HMGB1, and CRT. In addition, free cell staining was used to assess the expression and distribution of MHCII and CD74.

### 4.12 Ultrasound Imaging

Ultrasound and imaging were studied in vitro and in vivo. The contrast‐enhanced ultrasound imaging ability of mRNA/V@M NPs was evaluated using a contrast‐enhanced ultrasound instrument (LOGIQ E9, General Electric, USA). For in vitro ultrasound imaging, the mRNA/V@M NPs solution was added to the finger cover model of a latex glove and immersed in degassed water at 37°C before sonication. The main parameters are as follows: 9L linear transducer; Center frequency 9.0 MHz; Mechanical; The mechanical index (MI) was 0.8. The dynamic range was 60 dB. The focal length was 3.0 cm. The depth was 4.0 cm. In vivo, mice with fibrotic TNBC tumors were injected intravenously with mRNA/V@M NPs, ultrasound stimulation was added, and then ultrasound images of the tumors were obtained immediately. Tumor ultrasound images were acquired immediately after intravenous injection of 100 µL of mRNA/V@M NPs into tumor‐bearing mice. The main parameter is a 9 L linear transducer. Center frequency 9.0 MHz; MI 0.5; Dynamic range 48 dB; The focal point was 1 cm. The depth was 2.0 cm.

### 4.13 Biodistribution and In Vivo Organ Imaging

For biodistribution assays, mice with 4T1 (10^6^ cells) and NIH/3T3 (5 × 10^5^ cells) tumors were selected. DIR labeled mRNA/V@M NPs (100 µL) were injected via the tail vein. Then, the IVIS Spectral Imaging System (Perkin Elmer, USA) was used to acquire fluorescence images. At the predetermined time points (1, 2, 4, 8, and 24 h), mice were euthanized , and tumors and major organs were harvested for in vitro fluorescence imaging and quantitative biodistribution analysis. Tumors were collected to prepare tumor sections.

### 4.14 Immunohistochemistry(IHC)

For immunohistochemical analysis, tumor sections were first incubated overnight at 4 degrees with primary antibodies against Ki‐67, MHC II, CD74, S100A4, Fibronectin, PDGFR, αSMA, COL1A1, CD4, and CD8. Subsequently, secondary antibodies were applied and incubated at 37 degrees for 1 h. To assess apoptosis, a TUNEL staining assay was performed. A digital section scanning system (Olympus VS200) was used to visualize the expression of these proteins. Subsequently, ImageJ software was used to quantify the average fluorescence intensity, and relative expression levels were quantitatively assessed.

### 4.15 Detection of Immune Cells by FCM

Tumors, spleen, and lymphoid tissues were collected, ground, and passed through a 40micron filter to prepare single‐cell suspensions. The abundance of DC (CD11c^+^CD80^+^CD86^+^), CD8^+^T (CD3^+^CD8^+^) in fibrotic TNBC tumors and the abundance of CD8^+^ T (CD3^+^CD8^+^) in lymph nodes measured in fibrotic TNBC tumors were analyzed using flow cytometry (Beckman Coulter, Gallios). The abundance of regulatory T cells (Tregs, CD4^+^CD25^+^Foxp3^+^) in fibrotic TNBC tumors and spleens and the abundance of myeloid‐derived suppressor cells (MDSCs, CD11b^+^Gr1^+^) in fibrotic TNBC tumors were analyzed by flow cytometry. The distribution of effector memory T cells (TEM) (CD3^+^CD8^+^CD44^+^CD62L^−^) in the spleen was analyzed by flow cytometry. Antibodies used in the flow cytometry analysis were from Biolegend (Beijing, China). Data were processed using FlowJo.

### 4.16 Measurement of Cytokine Levels

Cytokines such as TNF‐α, IFN‐γ, and IL‐6 are considered to be key indicators of systemic immune activation. To measure the concentrations of TNF‐α, IFN‐γ, and IL‐6 cytokines, an ELISA kit (Bostek, China) was used. For in vitro experiments, 4T1 cells were inoculated into 6‐well plates and cultured for 24 h. After treatment with PBS, mRNA NPs, V‐9302 NPs, mRNA/V NPs, mRNA/V@M NPs, and mRNA/V@M+US (1 W/cm^2^, 60 s, 1 MHz) for 24 h, 4T1 cell cultures were collected separately, and ELISA kits (Bost, China) were used to determine the concentration of IFN‐β in the supernatants. To assess the maturation of dendritic cells in vitro, bone marrow‐derived dendritic cells (BMDCs) were extracted from BALB/c mice. BMDCs were stimulated with GM‐CSF (20 ng/mL) and IL‐4 (20 ng/mL). Immature dendritic cells were collected on day 7 and cultured for 24 h with 4T1 cell culture supernatant after group treatment. Culture supernatants were collected and measured using an ELISA kit.

For in vivo experiments, after group treatment, mice bearing tumors were euthanized, and their tumor samples were collected. Then, an appropriate amount of tumor tissue was mashed with an appropriate amount of saline, centrifuged at 1000 × g for 10 min, and the supernatant was taken to prepare for ELISA analysis. The OD value of each well was measured at 450 nm.

### 4.17 T Cell Activation Assays

An integrated co‐culture system consisting of myCAFs, DC2.4 cells, 4T1 tumor cells, and T cells was established to assess T cell activation after different nanoparticle treatments. Briefly, NIH3T3 fibroblasts were stimulated with TGF‐β1 to generate myCAF, then co‐cultured with 4T1 cells. They were treated with different groups. The treatment groups were as follows: G1, Control; G2, mRNA NPs; G3, V‐9302 NPs; G4, mRNA/V NPs; G5, mRNA/V@M NPs; and G6, mRNA/V@M NPs + US (1 W/cm^2^, 60 s, 1 MHz). After treatment, CAFs and 4T1 cells were co‐cultured with DC2.4 cells and T cells in complete RPMI 1640 medium. To assess the proliferation of CD4^+^ and CD8^+^ T cells, CD3^+^ T cells were isolated from the mouse spleen using the Mouse CD3^+^ T Cell Isolation Kit (Goonie; cat. no. 130‐6001). Unless otherwise stated, CAFs, 4T1 cells, DC2.4 cells, and T cells were seeded at a ratio of 1:1:1:5 and co‐cultured for 72 h at 37°C under 5% CO_2_. After co‐culture, suspended immune cells were collected for flow cytometric analysis. CD8^+^ T cell activation was evaluated by quantifying CD3^+^CD8^+^, CD3^+^CD8^+^Ki67^+^, and CD3^+^CD8^+^GZMB^+^ T cell populations. CD4^+^ T cell activation was assessed by analyzing CD3^+^CD4^+^ and CD3^+^CD4^+^Ki67^+^ T cells where indicated.

MHC I and MHC II blocking assays were performed to determine whether T cell activation was mediated through MHC I and MHC II dependent antigen‐presentation pathways. For the MHC II‐CD4^+^ T cell axis, myCAFs were treated with CD74 mRNA‐loaded formulations to induce apCAF reprogramming. After treatment, cells were washed with PBS and co‐cultured with T cells in complete RPMI 1640 medium. The groups were: G1, myCAFs + T cells; G2, CD74 mRNA‐treated myCAFs + T cells; and G3, CD74 mRNA‐treated myCAFs + T cells + anti‐MHC II antibody. The myCAFs and T cells were co‐cultured at a ratio of 1:4 for 72 h. For MHC II blockade, CD74 mRNA‐treated myCAFs were pre‐incubated with anti‐MHC II antibody at 10 µg/mL for 30 min before T cell seeding. Suspended T cells were then collected for flow cytometry. CD4^+^ T cell activation and proliferation were determined by the percentages of CD3^+^CD4^+^ and CD3^+^CD4^+^Ki67^+^ T cells.

For the MHC I‐CD8^+^ T cell axis, 4T1 cells were treated with V‐9302‐loaded formulations to induce ICD‐associated immunogenic activation. After treatment, 4T1 cells were washed with PBS and co‐cultured with DCs and T cells in complete RPMI 1640 medium. The groups were: G1, 4T1 cells + DCs + T cells; G2, V‐9302‐treated 4T1 cells + DCs + T cells; and G3, V‐9302‐treated 4T1 cells + DCs + T cells + anti‐MHC I antibody. V‐9302‐treated 4T1 cells were co‐incubated with DCs and T cells at a 4T1: DC: T cell ratio of 1:1:5 for another 72 h. For MHC I blockade, cells were pre‐incubated with anti‐MHC I antibody at 10 µg/mL for 30 min before T cell seeding. CD8^+^ T cell activation and proliferation were assessed by the percentages of CD3^+^CD8^+^ and CD3^+^CD8^+^Ki67^+^ T cells.

To evaluate the cooperative contribution of CAF reprogramming and ICD‐mediated tumor‐cell immunogenicity, a combination co‐culture system consisting of myCAFs, 4T1 cells, DCs, and T cells was established. The experimental groups were as follows: G1, myCAFs + 4T1 cells + DCs + T cells; G2, CD74 mRNA‐treated myCAFs + 4T1 cells + DCs + T cells; G3, myCAFs + V‐9302‐treated 4T1 cells + DCs + T cells; and G4, CD74 mRNA‐treated myCAFs + V‐9302‐treated 4T1 cells + DCs + T cells. After treatment, myCAFs and 4T1 cells were co‐cultured with DCs and T cells in complete RPMI 1640 medium. The myCAFs, 4T1 cells, DCs, and T cells were seeded at a ratio of 1:1:1:5 and cultured for 72 h. After co‐culture, non‐adherent immune cells were collected for flow cytometric analysis. CD4^+^ and CD8^+^ T cell responses were evaluated by quantifying CD3^+^CD4^+^, CD3^+^CD4^+^Ki67^+^, CD3^+^CD8^+^, and CD3^+^CD8^+^Ki67^+^ T cell populations.

### 4.18 Statistical Analysis

All quantitative data are presented as mean ± SD from at least three independent biological replicates, unless otherwise stated. Statistical analyses were performed using GraphPad Prism. Comparisons between two groups were analyzed using an unpaired two‐tailed Student's *t*‐test. Comparisons among multiple groups were performed using one‐way ANOVA followed by an appropriate post hoc test. A value of *p* < 0.05 was considered statistically significant. Statistical significance was indicated as ^*^ *p* < 0.05, ^**^ *p* < 0.01, ^***^ *p* < 0.001, and ^****^ *p* < 0.0001).

## Author Contributions

**Chen Ai**: investigation, conceptualization, methodology, writing – original draft. **Weikai Sun**: validation, writing – review and editing. **Yuxuan Zhao**: data curation, visualization. **Daqian Sun**: software. **Ting Meng**: formal analysis. **Yafei Qi**: supervision. **Fengyang Jiang**: software. **Jintang Sun**: formal analysis. **Zhiliang Gao**: supervision. **Dexin Yu**: supervision, resources, project administration, funding acquisition.

## Conflicts of Interest

The authors declare no conflicts of interest.

## Supporting information




**Supporting File**: advs76673‐sup‐0001‐SuppMat.docx.

## Data Availability

The data that support the findings of this study are available from the corresponding author upon reasonable request.
