# Supplementary material for: Spatiotemporally Ultrasound‐Controlled Nanoparticles Reprogramming Immunostimulatory Antigen‐Presenting Cancer‐Associated Fibroblasts to Enhance Cancer Immunotherapy
Source: Adv Sci (Weinh). 2026 Jul 27:e76673. Online ahead of print. doi: 10.1002/advs.76673 (PMC13403891; doi:10.1002/advs.76673)
Supplement: Supplementary file 1 — Supporting File: advs76673‐sup‐0001‐SuppMat.docx. [file ADVS-9999-e76673-s001.docx]

**Supporting Information**

**Spatiotemporally ultrasound-controlled nanoparticles reprogramming immunostimulatory antigen-presenting cancer-associated fibroblasts to enhance cancer immunotherapy**

Chen Ai ^1, 2, 3#^, Weikai Sun^1, 2, 3^, Yuxuan Zhao^1, 2, 3^, Daqian Sun^1, 2, 3^, Ting Meng^1, 2, 3^, Yafei Qi^1, 2^, Fengyang Jiang^1, 2^ Jintang Sun^3^, Zhiliang Gao^4,5,6^, Dexin Yu ^1, 2*^

^1^Department of Radiology, Qilu Hospital of Shandong University, Jinan, Shandong 250012, China

^2^Translational Medicine Research Center in Nano Molecular and Functional Imaging of Shandong University, Jinan, 250100, China

^3^Research Center for Basic Medical Sciences, Qilu Hospital of Shandong University, Jinan, China

^4^Shandong Key Laboratory of Magnetic Field-free Medicine & Functional Imaging, Shandong University, Jinan, Shandong 250012, China

^5^Research Institute of Magnetic Field-free Medicine & Functional Imaging, Shandong University, Jinan, Shandong 250012, China

^6^National Medicine-Engineering Interdisciplinary Industry-Education Integration Innovation Platform, Shandong University, Jinan, Shandong 250012, China

^#^These authors contributed equally to this work.

^*^To whom correspondence should be addressed at:

Dexin Yu, E-mail: yudexin0330@sina.com

Figure S1-S21


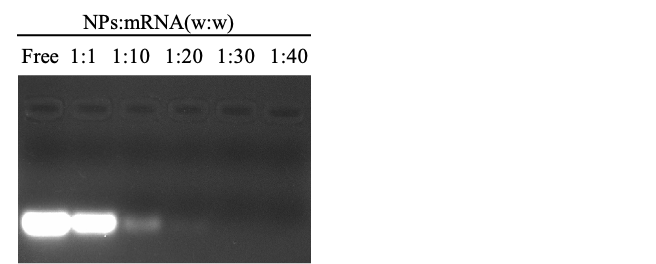


Figure S1. Agarose gel electrophoresis retardation assay of NPs-mRNA at various mass ratios.


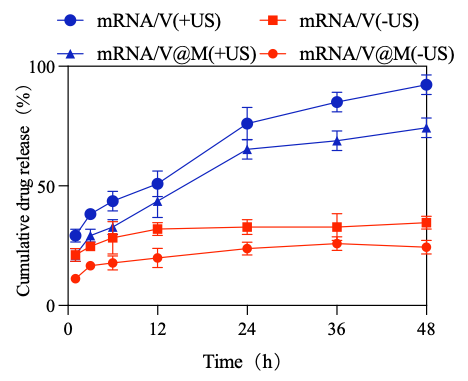


Figure S2. Drug release of mRNA/V and mRNA/V@M under the presence or absence of ultrasound stimulation.


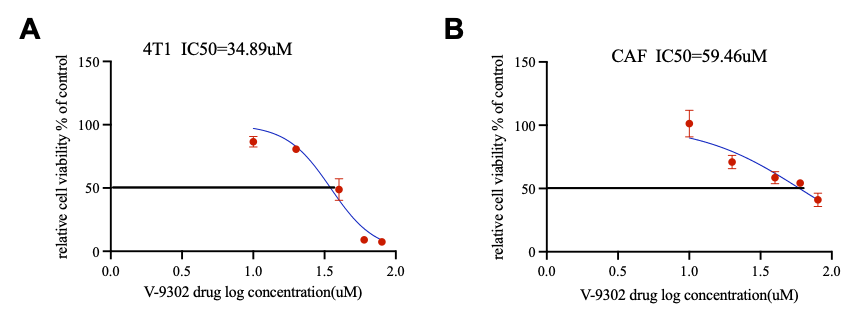


Figure S3. Cell viability of (A) 4T1 cells and (B) CAFs cells treated with different free V-9303 concentrations (*n* = 3).


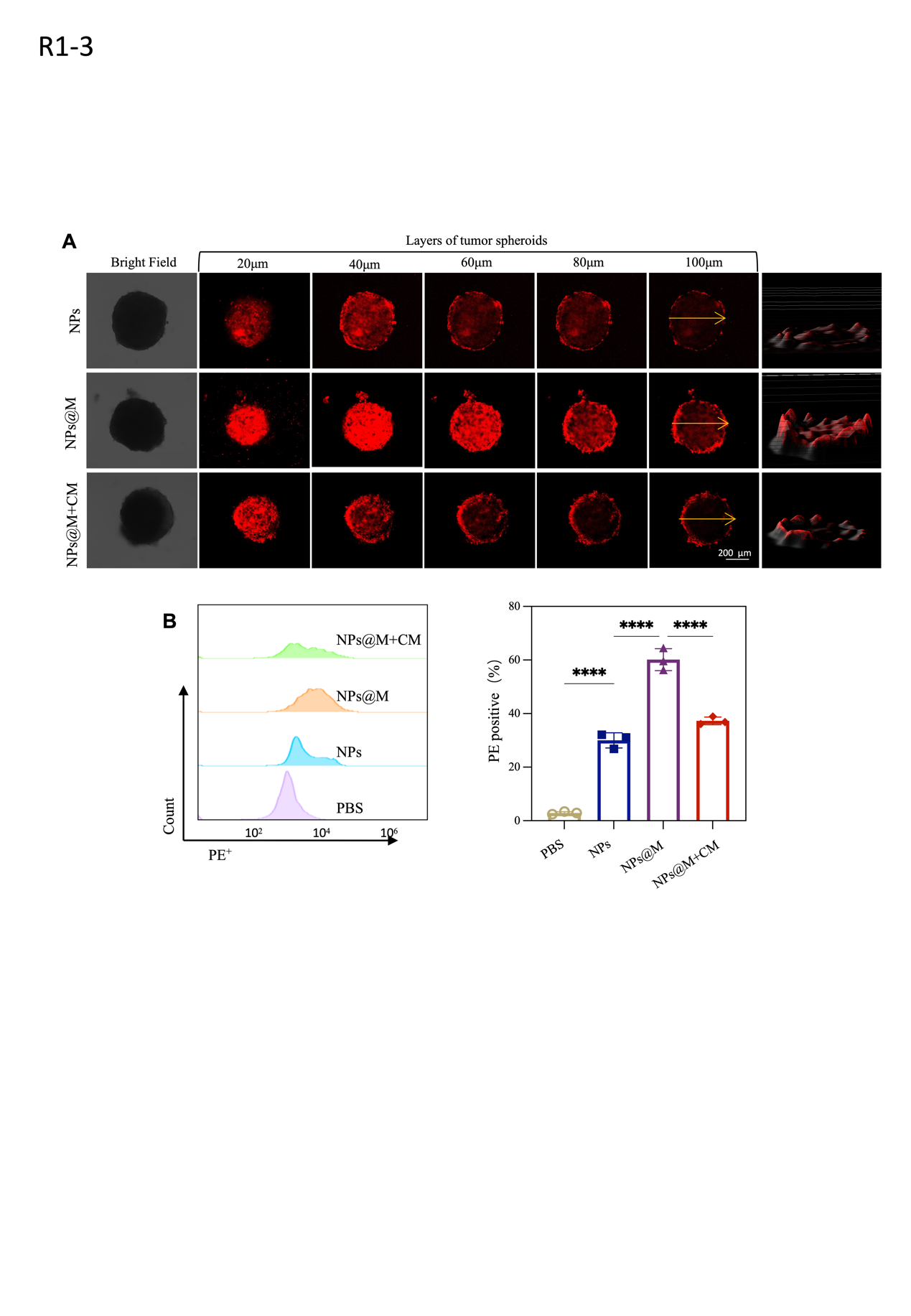


Figure S4. (A) Penetration and distribution of fluorescently labeled NPs, NPs@M, and NPs@M +CM (CAF membrane) in 3D tumor spheroids. 4T1 and CAFs were cultured in a 2:1 mixture. Scale bars, 200 μm. (B) Cellular uptake of fluorescently labeled NPs, NPs@M, and NPs@M +CM in CAFs. Data are presented as mean ± SD, n = 3. Statistical significance was determined by one-way ANOVA. **p* < 0.05, ***p* < 0.01, ****p* < 0.001, and *****p* < 0.0001.


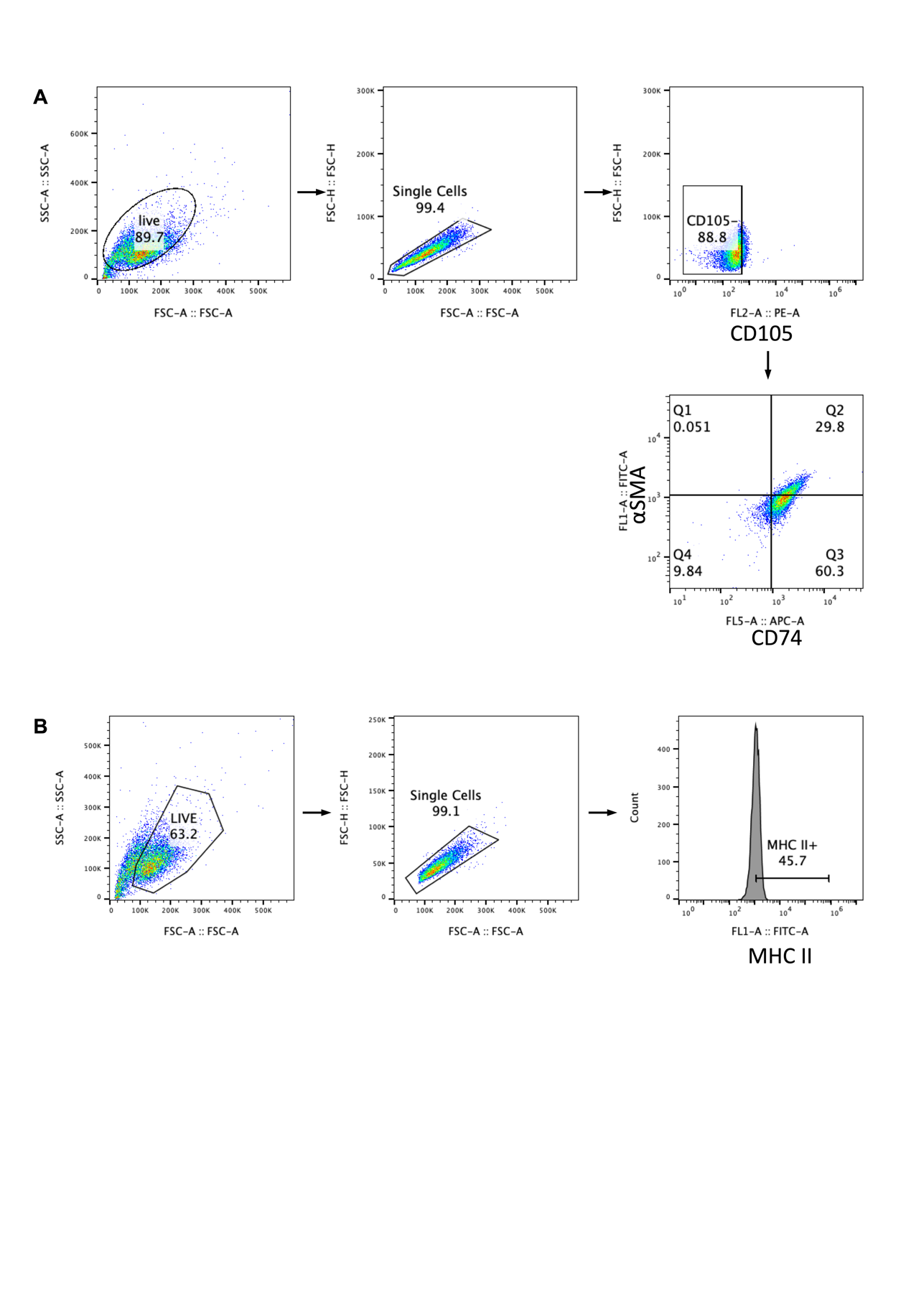


Figure S5. (A)Gating strategy for the expression of apCAFs markers (CD105^-^αSMA^-^CD74^+^) in TGFβ1 pre-activated NIH3T3 fibroblasts. (B) Gating strategy for MHC II expression in TGFβ1 pre-activated NIH3T3 fibroblasts.


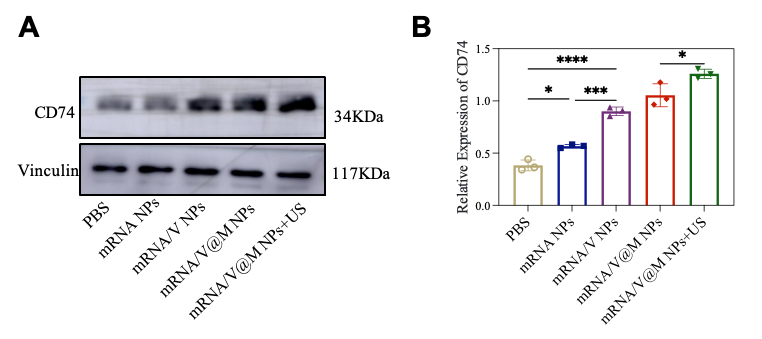


Figure S6. (A) Western blot analysis of CD74 in myCAFs with different treatments. Quantitative analysis of (B) CD74 expression in myCAFs (*n =* 3). Results were shown as mean ± SD. * *p* < 0.05, ***p* < 0.01, ****p* < 0.001 and *****p* < 0.0001 (ANOVA test).


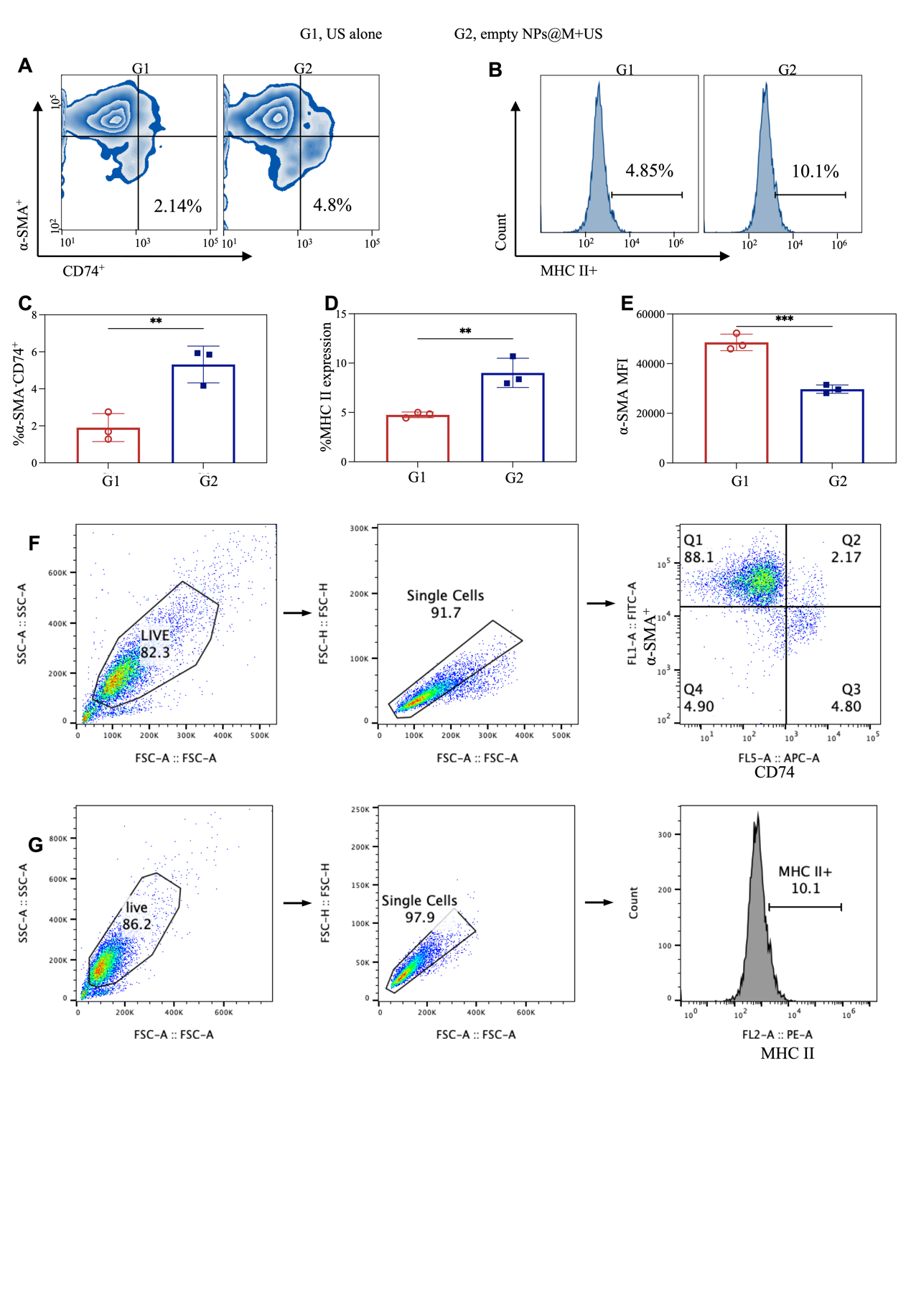


Figure S7. Evaluation of the independent contribution of ultrasound to CAF reprogramming.

Flow cytometric analysis was performed using additional control groups, including US alone and empty NPs@M + US, to distinguish the effect of ultrasound from that of the nanoparticle system.

(A) Representative flow cytometry plots of α-SMA⁻CD74⁺ CAFs. (B) Representative flow cytometry plots of MHC II⁺ CAFs. (C) Quantitative analysis of α-SMA⁻CD74⁺ CAFs. (D) Quantitative analysis of MHC II⁺ CAFs. (E) Quantitative analysis of α-SMA MFI in CAFs. (F)Gating strategy for the expression of apCAFs markers (αSMA^-^CD74^+^) in TGFβ1 pre-activated NIH3T3 fibroblasts. (G) Gating strategy for MHC II expression in TGFβ1 pre-activated NIH3T3 fibroblasts. Data are presented as mean ± SD, n = 3. Statistical significance was determined by *t*-test. **p* < 0.05, ***p* < 0.01, ****p* < 0.001 and *****p* < 0.0001.


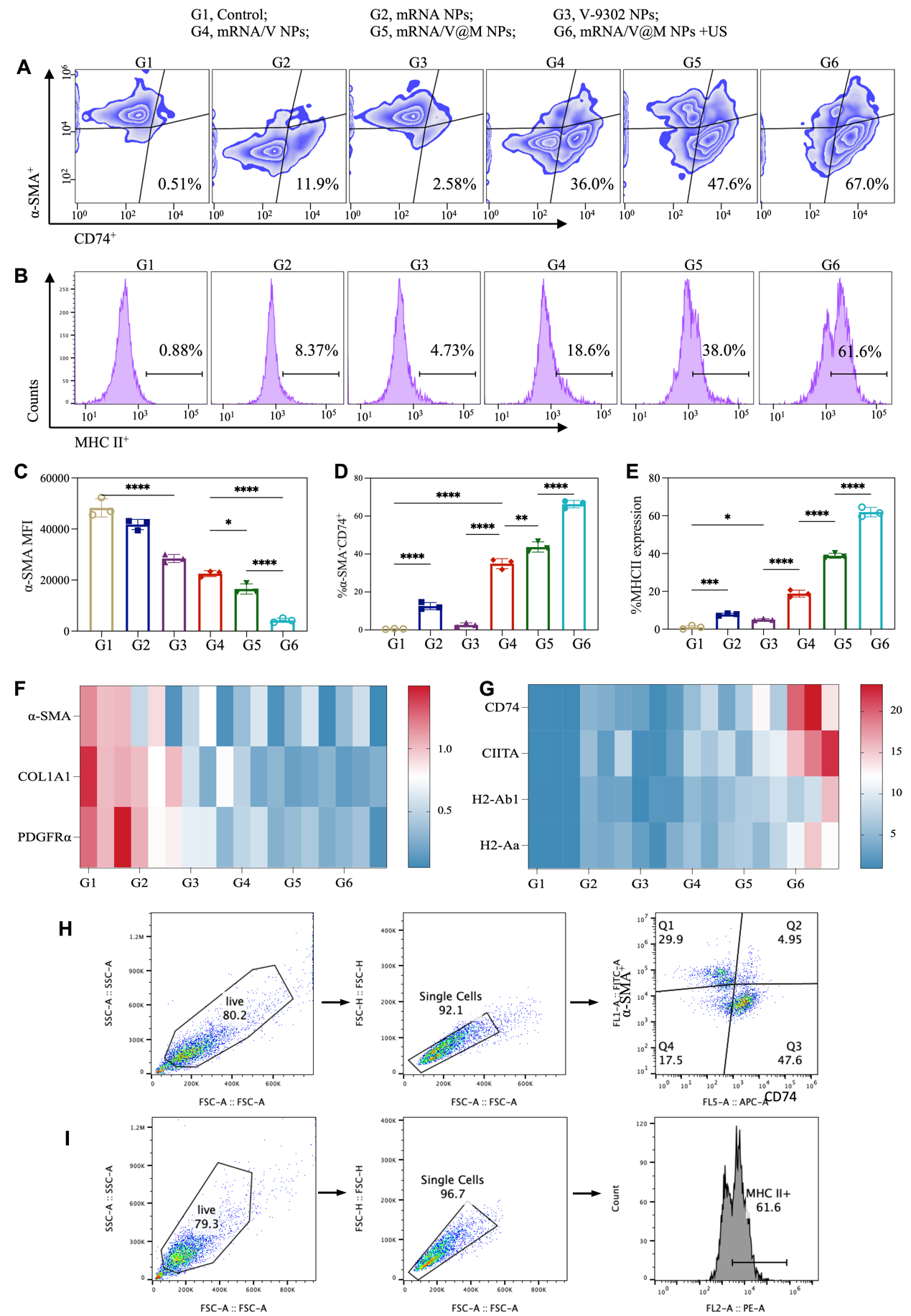


Figure S8. Validation of CAF reprogramming in primary CAFs. (A) Representative flow cytometry plots of α-SMA^-^CD74^+^ cells in primary CAFs after different treatments. (B) Representative flow cytometry plots of MHC II^+^ cells in primary CAFs after different treatments. (C) Quantitative analysis of α-SMA mean fluorescence intensity (MFI) in primary CAFs. (D) Quantitative analysis of the proportion of α-SMA^-^CD74^+^ cells. (E) Quantitative analysis of the proportion of MHC II^+^ cells. (F，G) Heatmap of qPCR analysis showing the expression of representative myCAF associated markers (α-SMA, PDGFR, and COL1A1)(F) and apCAF related markers (CD74, CIITA, H2-Ab1, and H2-Aa) (G)in primary CAFs after different treatments. The treatment groups included Group: G1, Control; G2, mRNA NPs; G3, V-9302 NPs; G4, mRNA/V NPs; G5, mRNA/V@M NPs; G6, mRNA/V@M NPs +US (1 W/cm^2^, 60 s, 1 MHz). (H) Gating strategy for the expression of apCAFs markers (αSMA^-^CD74^+^) in primary CAFs. (I) Gating strategy for MHC II expression in primary CAFs. Data are presented as mean ± SD, n = 3. Statistical significance was determined by one-way ANOVA. **p* < 0.05, ***p* < 0.01, ****p* < 0.001 and *****p* < 0.0001.


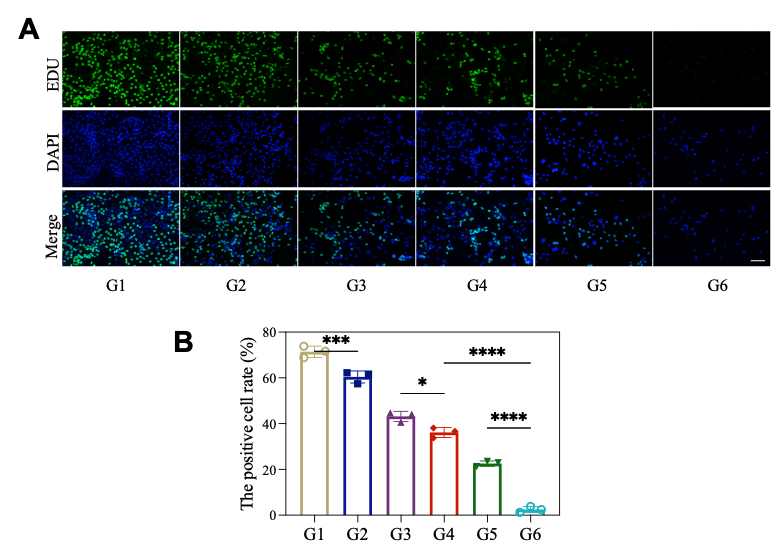


Figure S9. (A) Fluorescence images of EdU-positive cells after 24 h of various treatments. Scale bar: 200 μm.(B) Quantification results of EdU-positive cells assay. Group: G1, Control; G2, mRNA NPs; G3, V-9302 NPs; G4, mRNA/V NPs; G5, mRNA/V@M NPs; G6, mRNA/V@M+US (1 W/cm^2^, 60 s, 1 MHz). * *p* < 0.05, ***p* < 0.01, ****p* < 0.001 and *****p* < 0.0001 were statistically analyzed by One-way ANOVA.


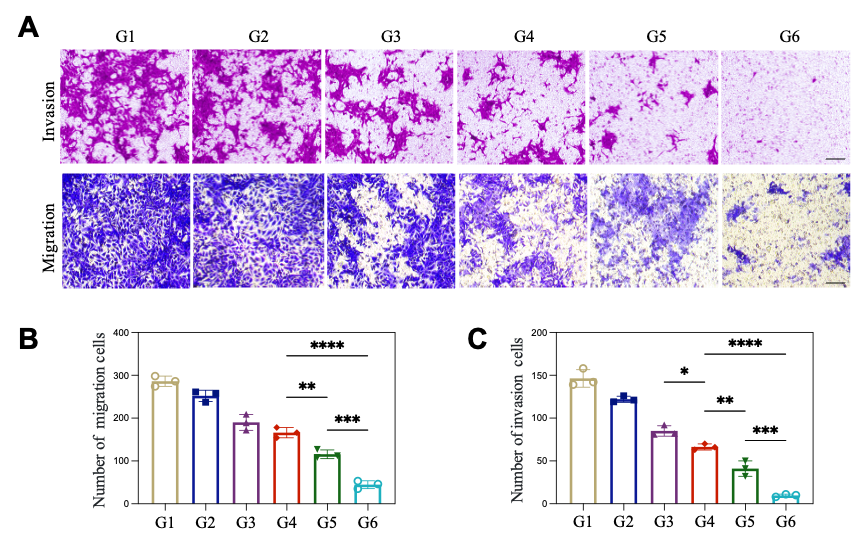


Figure S10. (A) *In vitro* cell invasion and migration experiments of 4T1 cells after different treatment. Scale bars: 200 μm. (B) Quantification results of cell invasion assay. *n =* 3. (C) Quantification results of cell migration assay. *n =* 3. Group: G1, Control; G2, mRNA NPs; G3, V-9302 NPs; G4, mRNA/V NPs; G5, mRNA/V@M NPs; G6, mRNA/V@M+US (1 W/cm^2^, 60 s, 1 MHz). * *p* < 0.05, ***p* < 0.01, ****p* < 0.001 and *****p* < 0.0001 were statistically analyzed by One-way ANOVA.


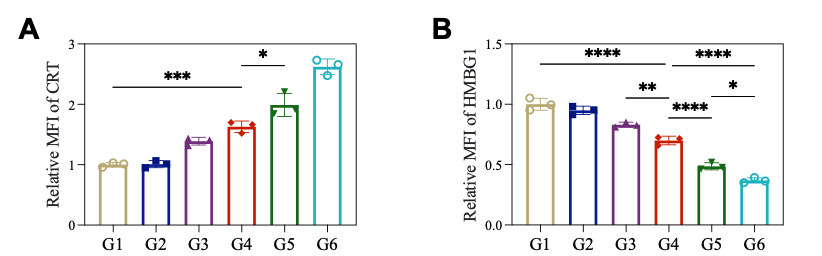


Figure S11. (A) Semi-quantification results of fluorescence intensity of CRT in different groups. (B) Semi-quantification results of fluorescence intensity of HMBG1 in different groups. Group: G1, Control; G2, mRNA NPs; G3, V-9302 NPs; G4, mRNA/V NPs; G5, mRNA/V@M NPs; G6, mRNA/V@M+US (1 W/cm2, 60 s, 1 MHz). * *p* < 0.05, ***p* < 0.01, ****p* < 0.001 and *****p* < 0.0001 were statistically analyzed by One-way ANOVA.


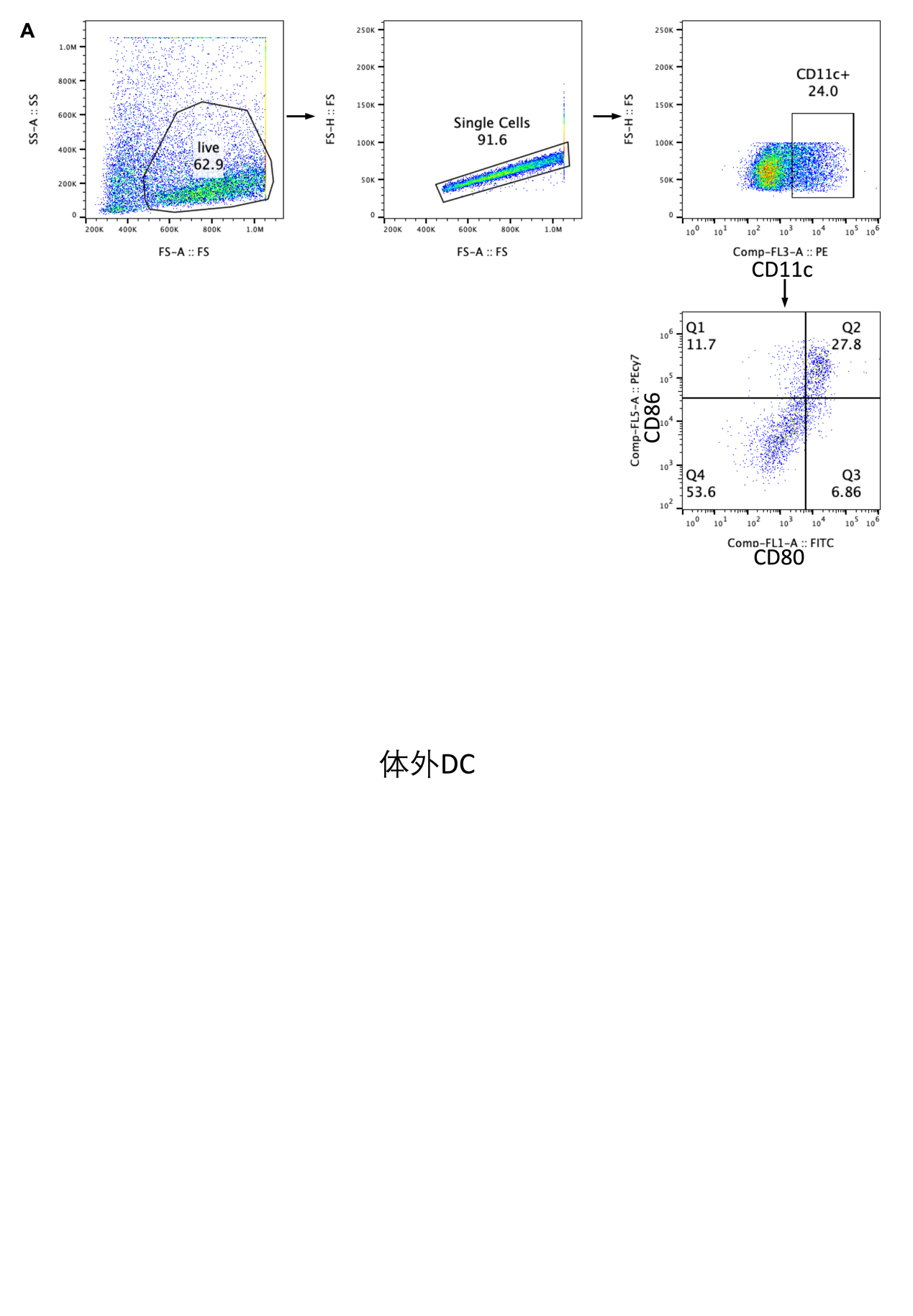


Figure S12. (A) Flow cytometry gating strategy of CD80⁺CD86⁺ DCs.


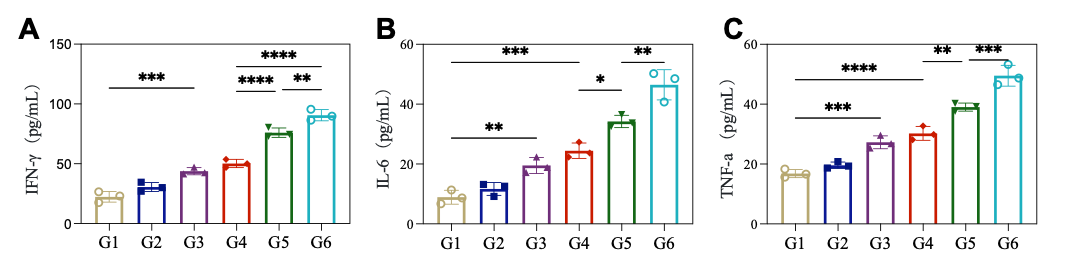


Figure S13. (A-C) Concentrations of IFN-β, IL-6, and TNF-α, measured by ELISA. Group: G1, Control; G2, mRNA NPs; G3, V-9302 NPs; G4, mRNA/V NPs; G5, mRNA/V@M NPs; G6, mRNA/V@M+US (1 W/cm2, 60 s, 1 MHz). * *p* < 0.05, ***p* < 0.01, ****p* < 0.001 and *****p* < 0.0001 were statistically analyzed by One-way ANOVA.


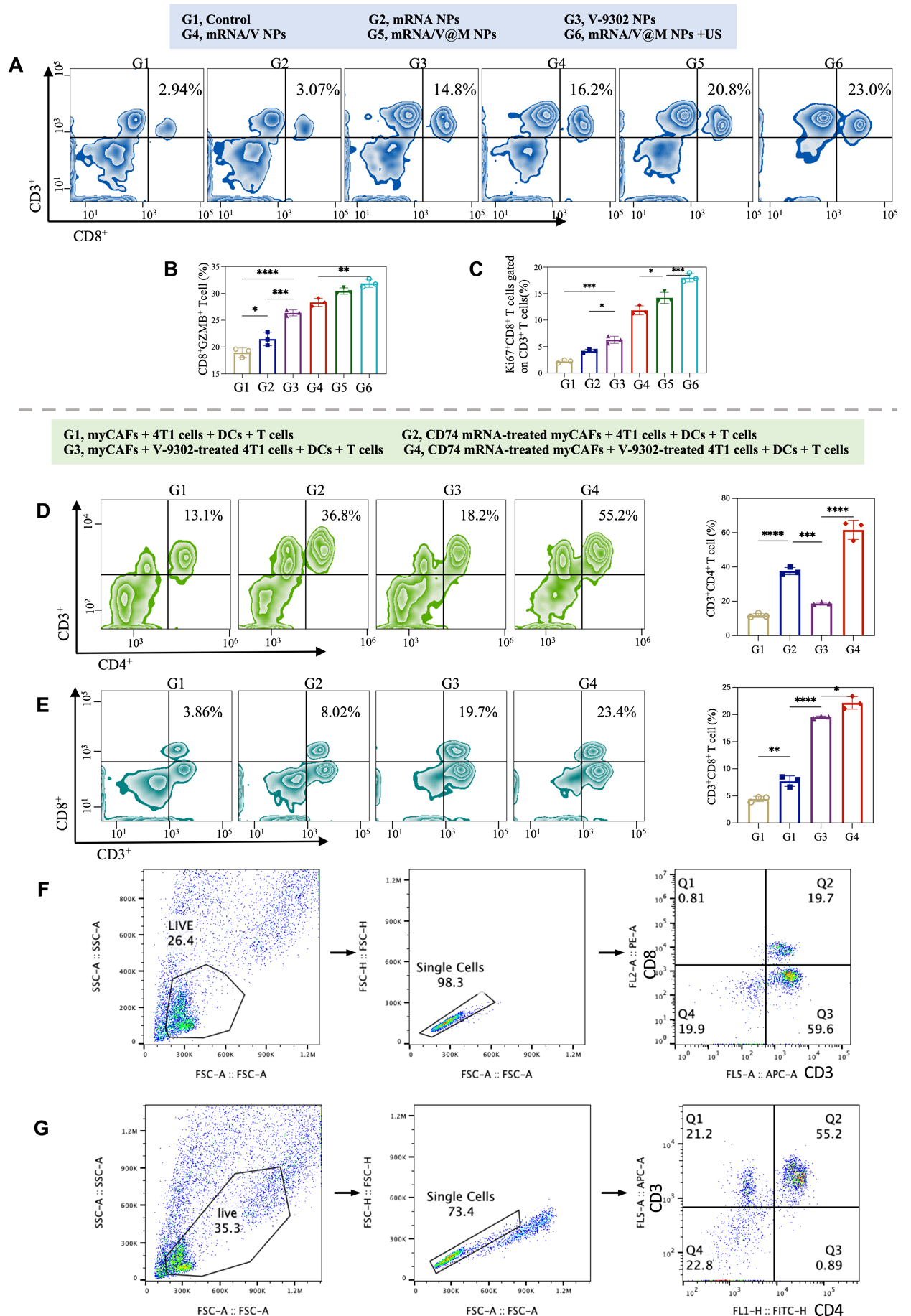


Figure S14. T cell activation and proliferation. (A) Representative flow cytometry plots of CD3⁺CD8⁺ cells after different treatments. Group: G1, Control; G2, mRNA NPs; G3, V-9302 NPs; G4, mRNA/V NPs; G5, mRNA/V@M NPs; G6, mRNA/V@M NPs +US (1 W/cm^2^, 60 s, 1 MHz). (B) Quantitative analysis of CD3⁺CD8⁺GZMB^+^ T cell. (C) Quantitative analysis of CD3⁺CD8⁺Ki67^+^ T cell. (D) Representative flow cytometry plots and quantitative analysis of CD3⁺CD4⁺ T cells in the integrated co-culture system consisting of myCAFs, 4T1 tumor cells, DCs, and T cells. (E) Representative flow cytometry plots and quantitative analysis of CD3⁺CD8⁺ T cells in the integrated co-culture system consisting of myCAFs, 4T1 tumor cells, DCs, and T cells. (F) Gating strategy for flow cytometry of CD3^+^CD4^+^ expression in CD4⁺ T cells. (G) Gating strategy for flow cytometry of CD3^+^CD8^+^ expression in CD8⁺ T cells. Data are presented as mean ± SD, n = 3. Statistical significance was determined by one-way ANOVA. **p* < 0.05, ***p* < 0.01, ****p* < 0.001 and *****p* < 0.0001.


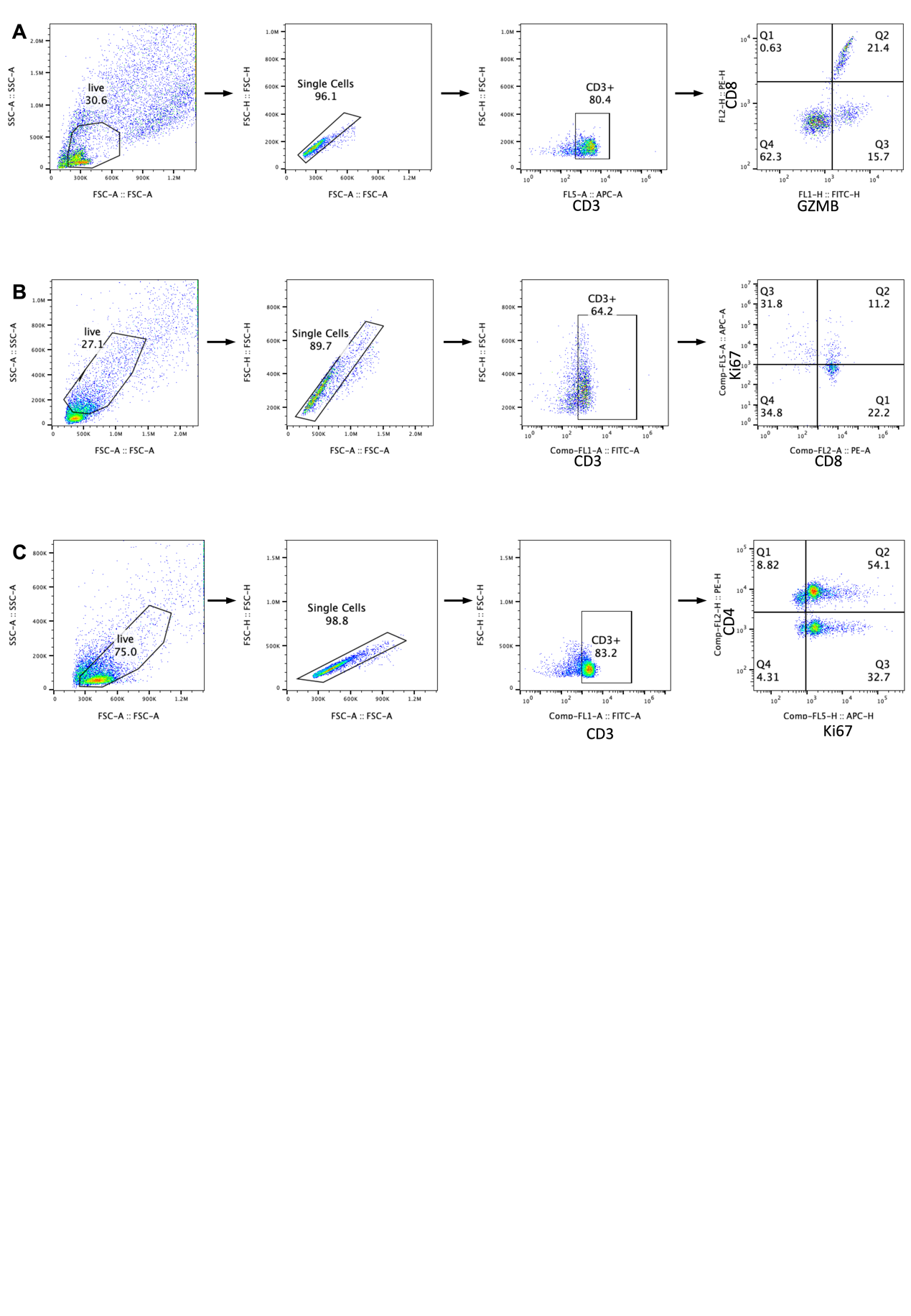


Figure S15. (A) Gating strategy for flow cytometry of CD3⁺CD8⁺GZMB⁺expression. (B) Gating strategy for flow cytometry of CD3⁺CD8⁺Ki67⁺ expression in CD8⁺ T cells. (C) Gating strategy for flow cytometry of CD3⁺CD4⁺Ki67⁺ expression in CD4⁺ T cells.


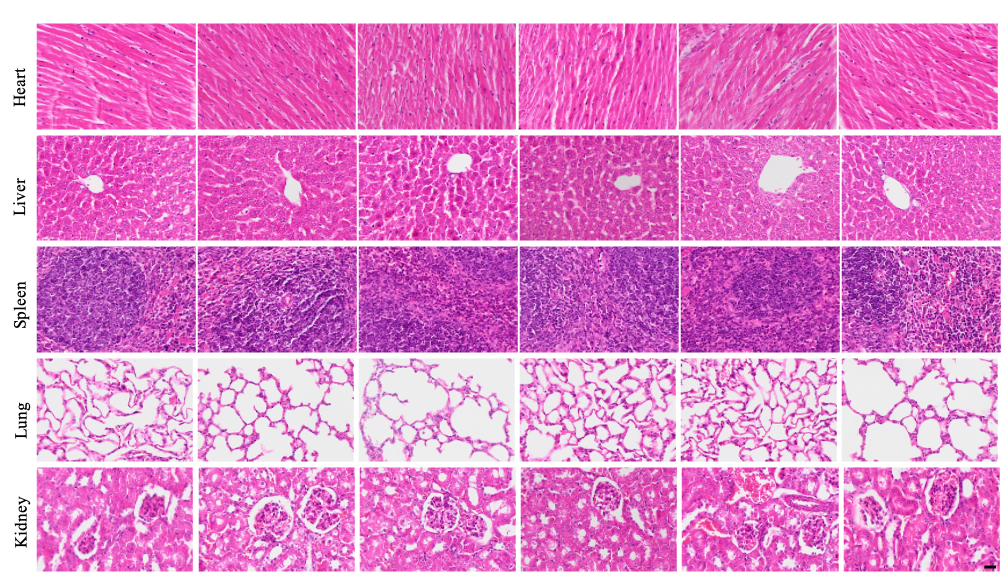


Figure S16. H&E staining images of the major organs. Scale bar: 20 µm.


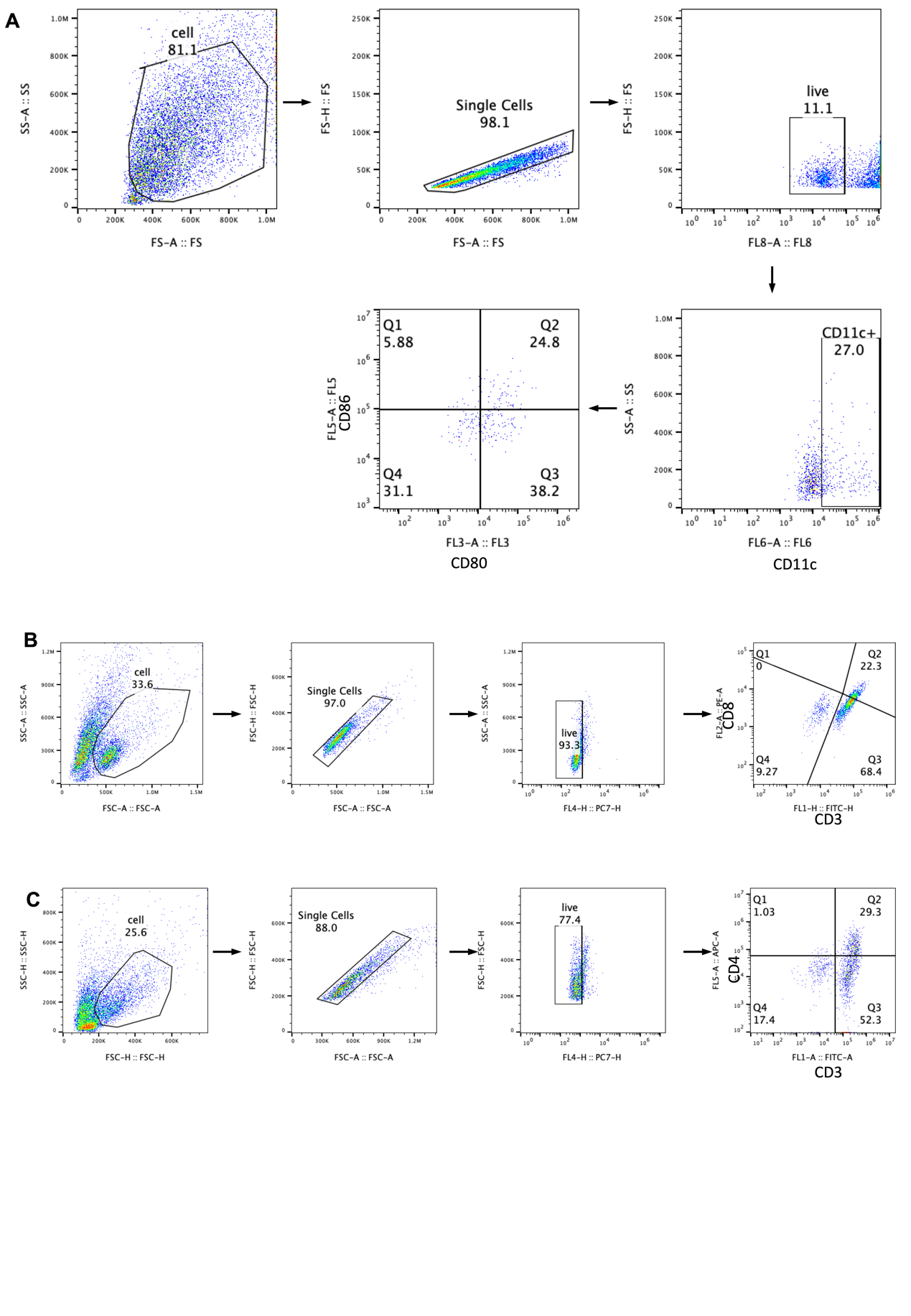


Figure S17. (A) Flow cytometry gating strategy of CD11c⁺CD86⁺CD80^+^ DCs in single-cell suspension of the tumor tissues. (B) Flow cytometry gating strategy of CD3⁺CD8⁺ T cells in single-cell suspension of the tumor tissues. (C) Flow cytometry gating strategy of CD3⁺CD4⁺ T cells in single-cell suspension of the tumor tissues.


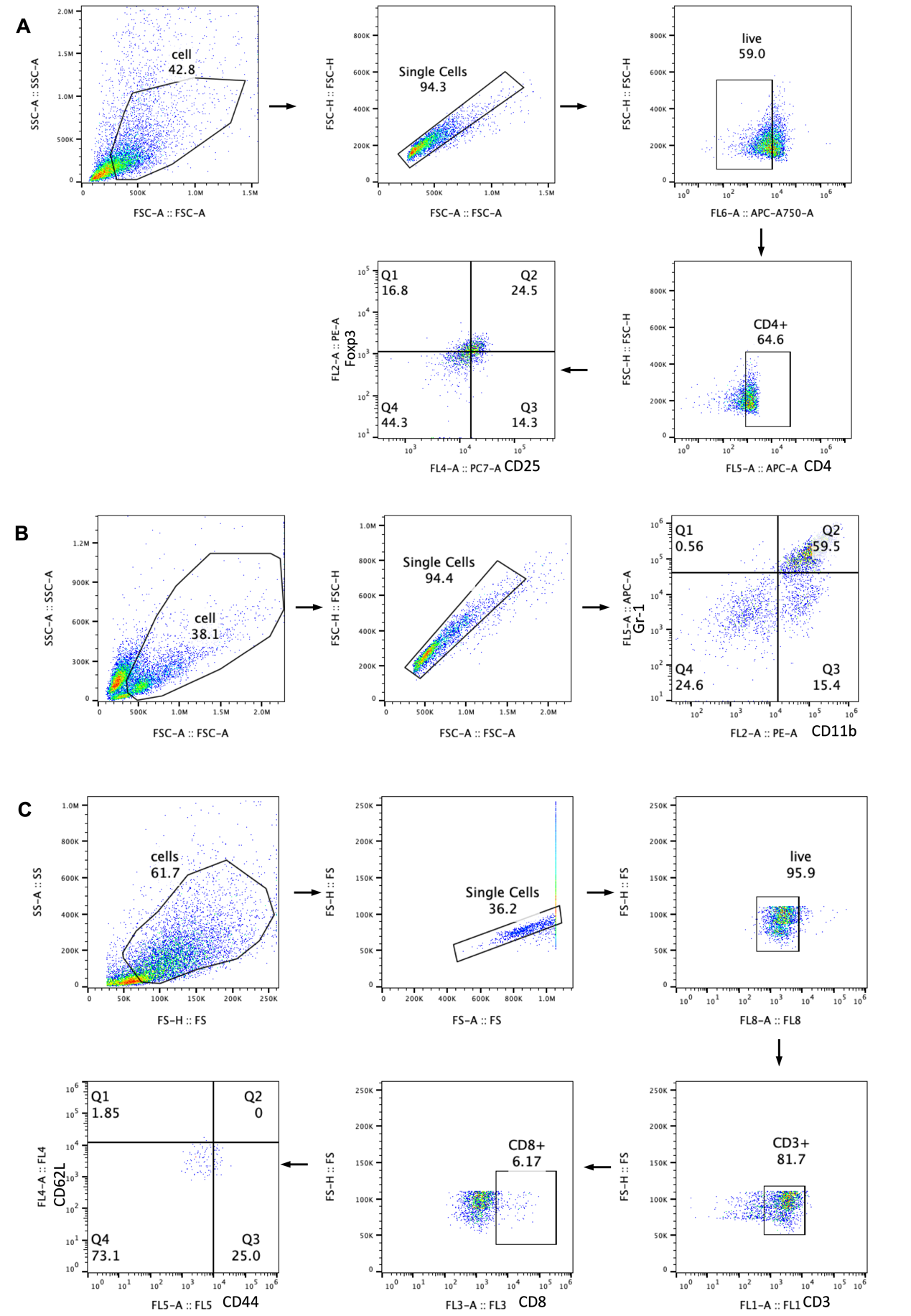


Figure S18. (A) Flow cytometry gating strategy of Tregs (CD4^+^CD25^+^FOXP3^+^) in single-cell suspension of the tumor tissues. (B) Flow cytometry gating strategy of MDSC (CD11b^+^Gr-1^+^) in single-cell suspension of the tumor tissues. (C) Flow cytometry gating strategy of T_EM_ (CD3^+^CD8^+^CD44^+^CD62L^−^) in the spleen.


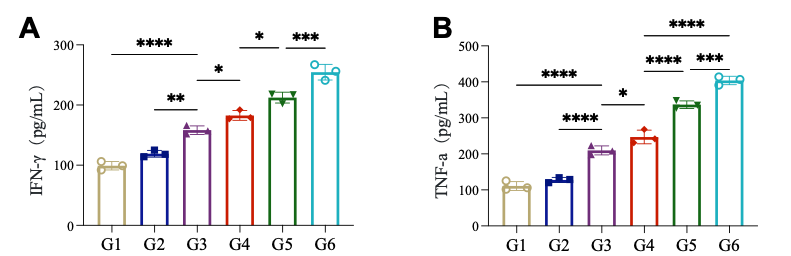


Figure S19. (A, B) Concentrations of IFN-γ and TNF-α in tumor tissues, measured by ELISA.


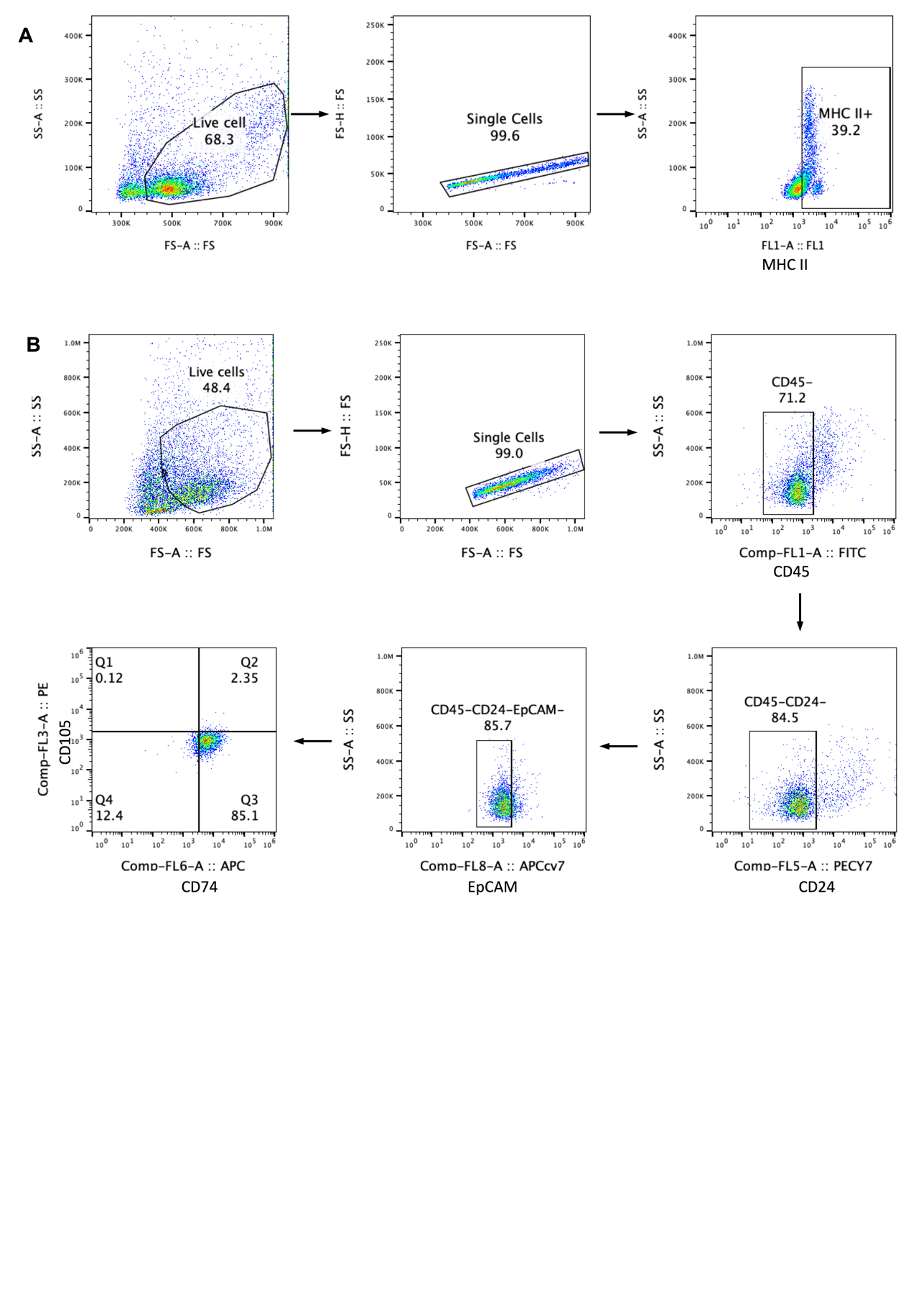


Figure S20. (A) Flow cytometry gating strategy of MHC II in single-cell suspension of the tumor tissues. (B) Flow cytometry gating strategy of CD45^-^CD24^-^EpCAM^-^CD105^-^CD74^+^ CAFs in single-cell suspension of the tumor tissues.


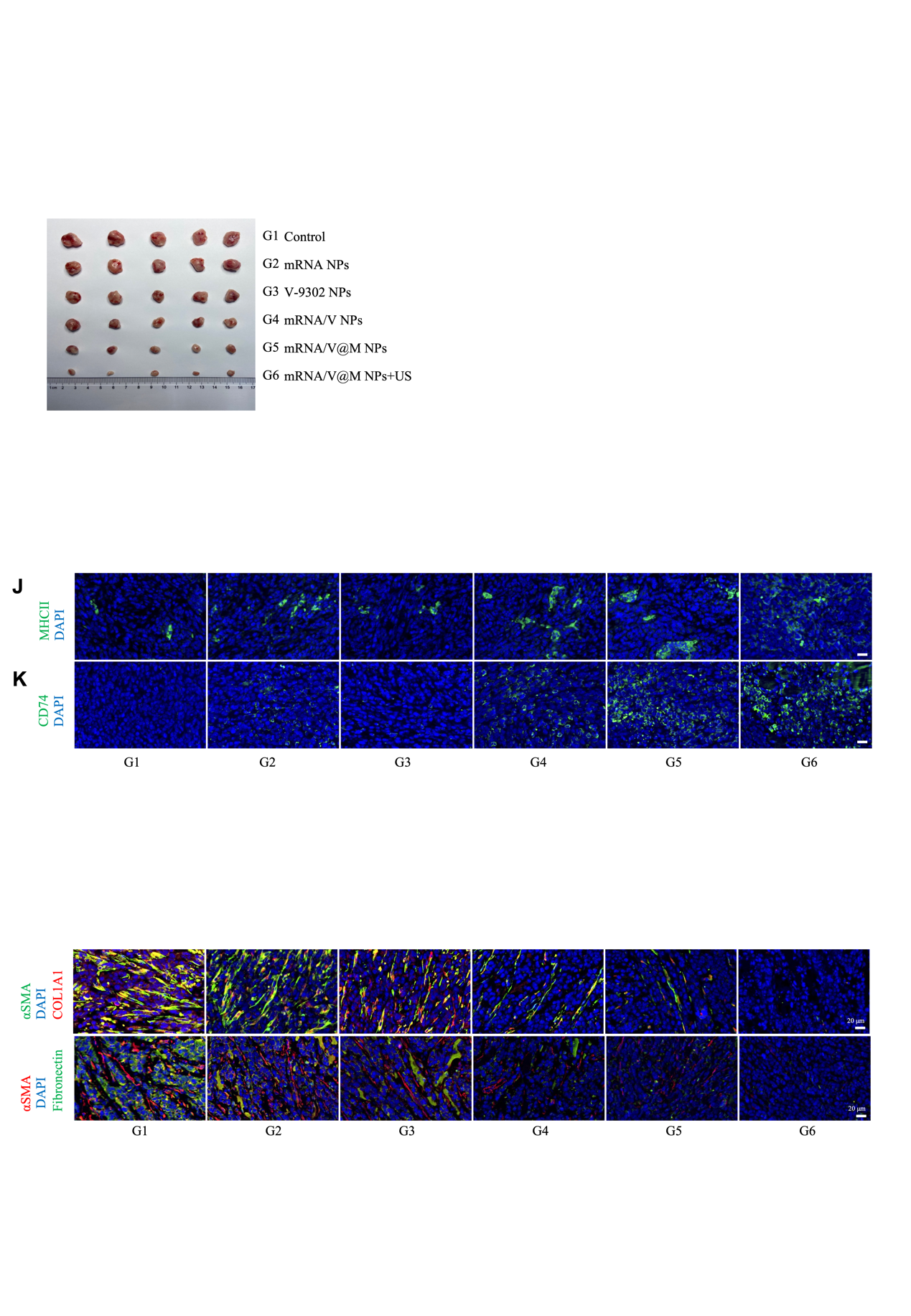


Figure S21. Representative images of Masson trichrome staining and immunofluorescence imaging of ECM markers (COL1A1 and Fibronection) in tumor sections after different treatments. Scale bars, 20 μm. Group: G1, Control; G2, mRNA NPs; G3, V-9302 NPs; G4, mRNA/V NPs; G5, mRNA/V@M NPs; G6, mRNA/V@M NPs+US (1 W/cm^2^, 60 s, 1 MHz).
